# Mitochondrial mistranslation modulated by metabolic stress causes cardiovascular disease and reduced lifespan

**DOI:** 10.1111/acel.13408

**Published:** 2021-06-07

**Authors:** Tara R. Richman, Judith A. Ermer, Stefan J. Siira, Irina Kuznetsova, Christopher A. Brosnan, Giulia Rossetti, Jessica Baker, Kara L. Perks, Henrietta Cserne Szappanos, Helena M. Viola, Nicola Gray, Mark Larance, Livia C. Hool, Steven Zuryn, Oliver Rackham, Aleksandra Filipovska

**Affiliations:** ^1^ Harry Perkins Institute of Medical Research QEII Medical Centre Nedlands WA Australia; ^2^ ARC Centre of Excellence in Synthetic Biology QEII Medical Centre Nedlands WA Australia; ^3^ Centre for Medical Research QEII Medical Centre, The University of Western Australia Nedlands WA Australia; ^4^ Clem Jones Centre for Ageing Dementia Research Queensland Brain Institute The University of Queensland Brisbane Qld Australia; ^5^ Telethon Kids Institute Perth Children's Hospital Nedlands WA Australia; ^6^ School of Pharmacy and Biomedical Sciences Curtin University Bentley WA Australia; ^7^ School of Human Sciences The University of Western Australia Nedlands WA Australia; ^8^ Australian National Phenome Centre Centre for Computational and Systems Medicine Health Futures Institute Murdoch University Perth WA Australia; ^9^ Charles Perkins Centre School of Life and Environmental Sciences University of Sydney Sydney NSW Australia; ^10^ Victor Chang Cardiac Research Institute Sydney NSW Australia; ^11^ Curtin Health Innovation Research Institute Curtin University Bentley WA Australia; ^12^ School of Molecular Sciences The University of Western Australia Perth WA Australia

**Keywords:** ageing, metabolism, mitochondria, protein synthesis

## Abstract

Changes in the rate and fidelity of mitochondrial protein synthesis impact the metabolic and physiological roles of mitochondria. Here we explored how environmental stress in the form of a high‐fat diet modulates mitochondrial translation and affects lifespan in mutant mice with error‐prone (*Mrps12^ep^* ^/^ *^ep^*) or hyper‐accurate (*Mrps12^ha^* ^/^ *^ha^*) mitochondrial ribosomes. Intriguingly, although both mutations are metabolically beneficial in reducing body weight, decreasing circulating insulin and increasing glucose tolerance during a high‐fat diet, they manifest divergent (either deleterious or beneficial) outcomes in a tissue‐specific manner. In two distinct organs that are commonly affected by the metabolic disease, the heart and the liver, *Mrps12^ep^* ^/^ *^ep^* mice were protected against heart defects but sensitive towards lipid accumulation in the liver, activating genes involved in steroid and amino acid metabolism. In contrast, enhanced translational accuracy in *Mrps12^ha^* ^/^ *^ha^* mice protected the liver from a high‐fat diet through activation of liver proliferation programs, but enhanced the development of severe hypertrophic cardiomyopathy and led to reduced lifespan. These findings reflect the complex transcriptional and cell signalling responses that differ between post‐mitotic (heart) and highly proliferative (liver) tissues. We show trade‐offs between the rate and fidelity of mitochondrial protein synthesis dictate tissue‐specific outcomes due to commonly encountered stressful environmental conditions or aging.

## Graphical abstract

This work reveals how trade‐offs between the rate and fidelity of mitochondrial protein synthesis dictate tissue‐specific outcomes to metabolic stress or aging. Error‐prone protein synthesis enhanced the rate of translation, protecting against heart defects but stimulated lipid accumulation in the liver. Enhanced translational accuracy protected liver function through activation of proliferation programs, but resulted in severe hypertrophic cardiomyopathy and reduced lifespan.

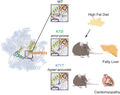

## 1 INTRODUCTION

Protein synthesis is essential for cell function and it can take place in the cytoplasm and also in organelles such as mitochondria and chloroplasts. Protein synthesis involves an orchestrated readout of mRNAs by ribosomes, facilitated by translation factors and tRNAs, however, this process can be remarkably error‐prone (Schwartz & Pan, 2017). Amino acid misincorporation can occur in every 10^3^–10^4^ mRNA codons during translation and it has been suggested that ~15% of all proteins may have at least one misincorporated amino acid (Ogle & Ramakrishnan, 2005; Parker & Friesen, 1980). Cells can function effectively in spite of translational errors due to the efforts of quality control mechanisms and the ability to tolerate a low level of non‐programmed amino acid incorporation in prokaryotes and eukaryotes (Wang et al., 2006; Wang & Pan, 2016).

Translational efficiency balances the fidelity and speed of protein synthesis to ensure a functional compromise between protein yield and integrity. Altering the balance of translational efficiency can accrue costs to the growth and propagation of cells, as higher accuracy of translation is gained at the expense of speed and the mechanisms ensuring the accuracy of protein synthesis require higher energy use (Suhm et al., 2018). Errors in protein synthesis reduce the fitness of an organism by causing the loss of function of a protein caused by mistranslation, or production of polypeptides that have gained toxic functions. Under certain physiological and environmental conditions, different levels of translational accuracy and speed may be more or less tolerated (Schwartz & Pan, 2017). For example, mammalian cells can benefit from tRNA misacylation in certain environments such as upon exposure to toll‐like receptor ligands or chemically induced oxidative stress (Luo & Levine, 2009).

Defects in the fidelity of cytoplasmic protein synthesis and folding can reduce lifespan (Xie et al., 2019) or cause severe diseases, including neurodegeneration, cancer, heart disease, and viral infections (Brüning & Jückstock, 2015; Hekman et al., 2012; Lee et al., 2006; Liu et al., 2014; Netzer et al., 2009; Schimmel & Guo, 2009; Sulima et al., 2014). Impaired fidelity of mitochondrial translation also leads to diseases such as cardiomyopathy (Rudler et al., 2019). This is because the fidelity of mitochondrial protein synthesis is essential for the expression of the 13 respiratory complex polypeptides encoded by the mitochondrial genome and their coordinated assembly with the nuclear‐encoded proteins of the oxidative phosphorylation (OXPHOS) system (Rudler et al., 2019). Defects in mitochondrial protein synthesis are the most common causes of mitochondrial diseases, for which there are currently no cures (Boczonadi & Horvath, 2014). Therefore, it is important to gain better insight into the pathology of diseases caused by defects in the rate and fidelity of mitochondrial translation.

Error‐prone translation in yeast mitochondria results in ROS production and activation of a general stress response (Suhm et al., 2018), causing a deleterious response similar to that in bacteria (Agarwal et al., 2011). In *Caenorhabditis elegans* reduction of the mitochondrial ribosomal protein of the small subunit 5 (MRPS5), which can confer mistranslation, leads to reduced mitochondrial protein synthesis and increased lifespan (Liu et al., 2020; Molenaars et al., 2020). In mammals, an error‐prone mutation in MRPS12 was beneficial, causing upregulation of mitochondrial stress signalling and biogenesis to compensate for the reduced OXPHOS levels and function due to a mistranslation (Ferreira et al., 2019). A hyper‐accurate mutation in the same protein reduced the rate of mitochondrial protein synthesis without eliciting a rescue stress response (Ferreira et al., 2019), resulting in a persistent reduction of OXPHOS function that caused heart disease.

To explore the physiological consequence of the error‐prone and hyper‐accurate mutations in mammals compared to other systems we challenged mouse models with a metabolic stress and observed changes during ageing. We found that on a calorie‐rich diet the error‐prone mutation caused metabolic dysfunction and the hyper‐accurate mutation resulted in reduced lifespan in worms and mice. Our study reveals that dietary stress and ageing can have a detrimental impact on the fidelity and rate of protein synthesis and consequently energy metabolism.

## 2 RESULTS

### 2.1 High‐fat diet leads to reduced weight of mice with a mutation causing hyper‐accurate protein synthesis

**FIGURE 1:**
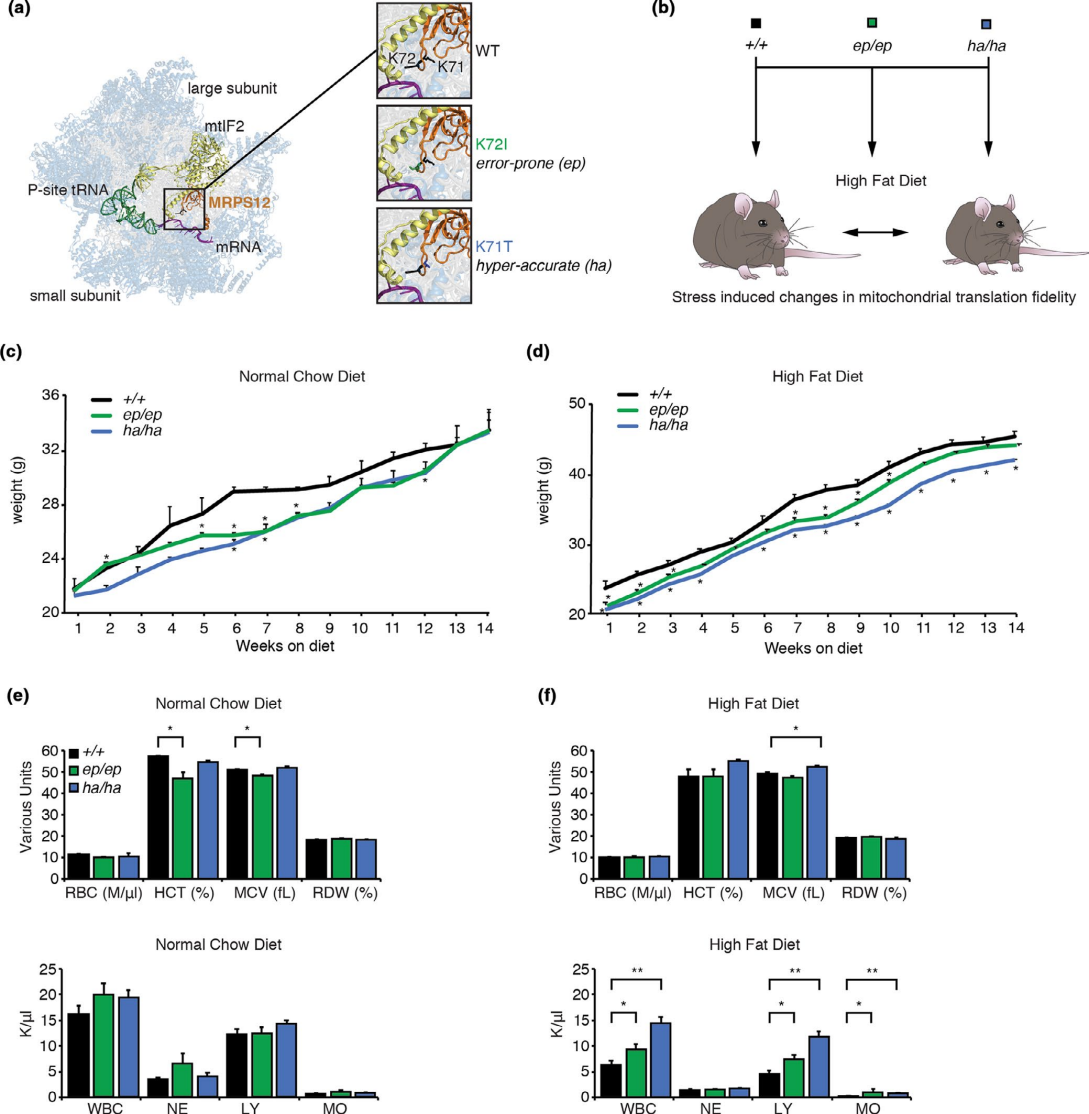
A high‐fat diet modulates the effects of error‐prone and hyper‐accurate mitochondrial translation in mice. (a) Mutations in the mitoribosomal decoding site protein, MRPS12, generate mice with altered fidelity of mitochondrial translation. MRPS12 (orange) is shown within the structure of the mitochondrial ribosome (PDB ID: 6GAW; Kummer et al., 2018). Ribosomal proteins are shown in blue and rRNAs in grey. The ribosome is rendered semi‐transparent to reveal MRPS12, P‐site fMet‐tRNAMet (green), mRNA (purple), and mtIF2 (yellow) in the decoding site. Inset images show close up views of the mutated residues at positions 71 and 72 of MRPS12, with K72I and K71T mutations modelled using PyMOL (Schrödinger). (b) Schematic representation of the experimental design. Wild‐type, error‐prone (*Mrps12^ep^* ^/^ *^ep^*) and hyper‐accurate (*Mrps12^ha^* ^/^ *^ha^*) mouse models are metabolically stressed when fed a high‐fat diet. Weight of *Mrps12* ^+/+^, *Mrps12^ep^* ^/^ *^ep^* and *Mrps12^ha^* ^/^ *^ha^* mice fed an NCD (c) and HFD (d) is shown from 6 to 20 weeks of age. Red blood cells (RBC), hematocrit (HCT), mean corpuscular volume (MCV), red cell distribution width (RDW) and white blood cell (WBC), neutrophil count (NE), lymphocyte count (LY), monocyte count (MO) were analysed using a Hemavet 950 Haematology System in *Mrps12* ^+/+^, *Mrps12^ep^* ^/^ *^ep^* and *Mrps12^ha^* ^/^ *^ha^* mice on an NCD (e) and HFD (f). All analyses were performed on at least six mice of each genotype and quantitative values are ±SEM. *p < 0.05, Students *t* test

The quality control requirements for protein synthesis differ between organisms and variability in decoding is at times evolutionarily conserved and advantageous (Moghal et al., 2014). Different mutations or environmental conditions have been shown to increase the error rate of protein synthesis (Gromadski & Rodnina, 2004; Lee et al., 2006). However, the molecular and physiological consequences of both environmental conditions and mutations that change the fidelity of protein synthesis have not been studied *in* *vivo* previously. Therefore, we used error‐prone, *Mrps12^ep^* ^/^ *^ep^*, and hyper‐accurate, *Mrps12^ha^* ^/^ *^ha^*, mouse models of protein synthesis that have either a K72I mutation or K71T mutation in MRPS12, respectively (Figure 1a; Ferreira et al., 2019), altering domain closing in the ribosome decoding centre and causing them to have increased or decreased amino acid misincorporation. To evaluate the effects of each of the mutations under different environmental conditions we fed them a normal chow diet (NCD) or high‐fat diet (HFD) from 6 weeks of age for 14 weeks and evaluated the physiological and molecular consequences (Figure 1b). *Mrps12^ep^* ^/^ *^ep^* and *Mrps12^ha^* ^/^ *^ha^* mice fed an NCD or HFD were weighed weekly to monitor changes in weight compared to control mice. The food intake of the control, *Mrps12^ep^* ^/^ *^ep^* and *Mrps12^ha^* ^/^ *^ha^* mice was the same over 14 weeks (Figure S1). Although the weight of the *Mrps12^ep^* ^/^ *^ep^* and *Mrps12^ha^* ^/^ *^ha^* mice was significantly reduced at weeks 5–8 and 12, by 14 weeks on an NCD the mutant mice had no differences in their weight compared to control mice (Figure 1c). On the HFD the *Mrps12^ep^* ^/^ *^ep^* mice had reduced weight at 6–10 weeks but their weight normalised compared to control mice. In contrast, the *Mrps12^ha^* ^/^ *^ha^* mice were significantly leaner compared to both wild type and *Mrps12^ep^* ^/^ *^ep^* mice throughout the HFD regime (Figure 1d), indicating that the hyper‐accurate mutation in MRPS12 results in a metabolic change and reduced body weight.

The blood cell profiles of the mice revealed that the *Mrps12^ep^* ^/^ *^ep^* mutant mice on an NCD have a significant reduction in haematocrit (HCT) and mean corpuscular (erythrocyte) volume (MCV), indicative of smaller blood cells and an iron deficiency (Figure 1e). In contrast, the MCV was increased in the *Mrps12^ha^* ^/^ *^ha^* mice fed an HFD (Figure 1f). White blood cell, lymphocyte and monocyte levels are comparable between the control and mutant mouse strains on an NCD (Figure 1e), however, when fed an HFD their levels were increased significantly in both mutant mouse strains compared to control mice (Figure 1f). In the *Mrps12^ha^* ^/^ *^ha^* mice the white blood cell and lymphocyte levels were much higher compared to both the control and *Mrps12^ep^* ^/^ *^ep^* mutant mice, indicating the presence of an immune response to inflammation.

### 2.2 High‐fat diet elicits tissue‐specific changes in response to altered fidelity of mitochondrial protein synthesis

**FIGURE 2:**
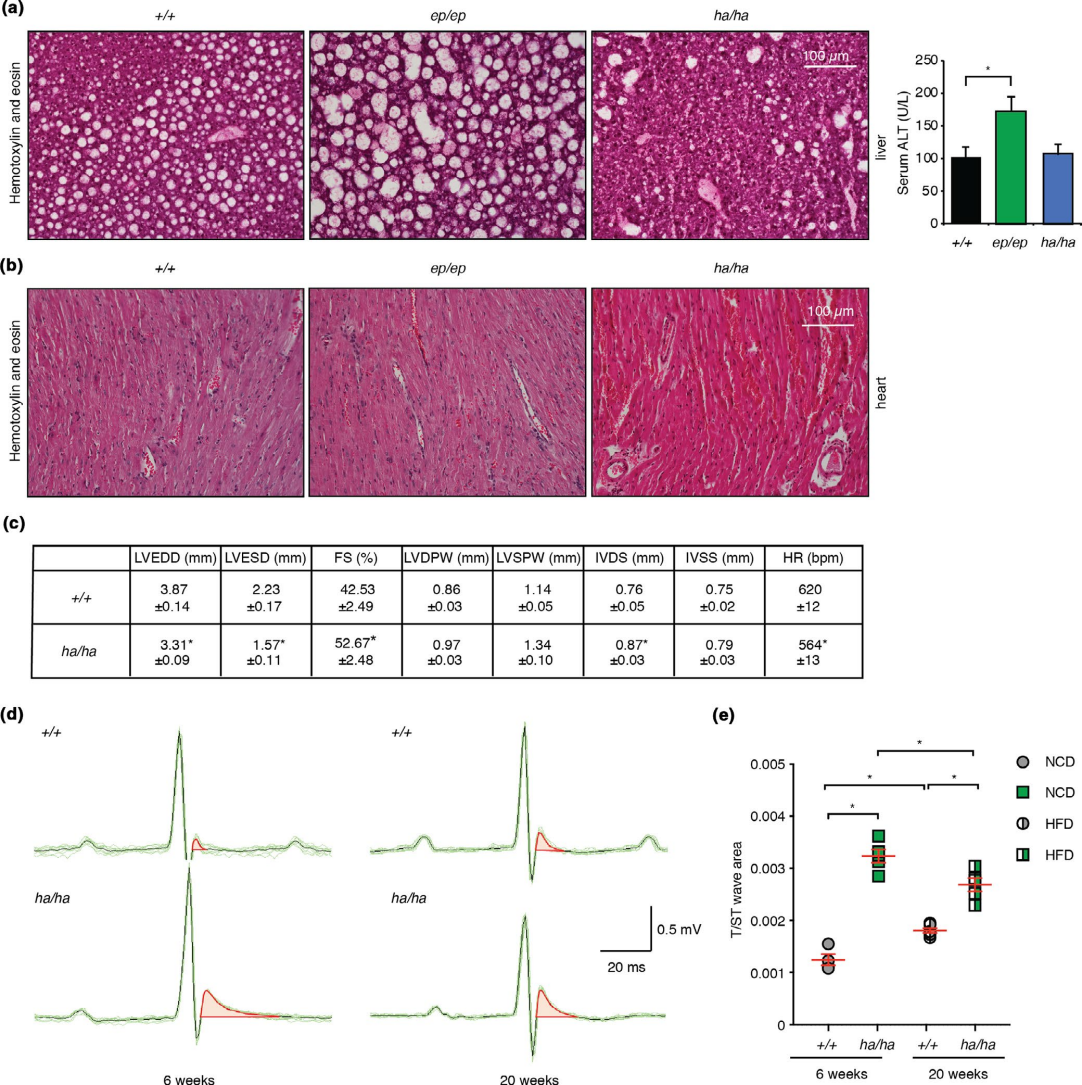
*Mrps12^ha^* ^/^ *^ha^* mice develop hypertrophic cardiomyopathy on a high‐fat diet and *Mrps12^ep^* ^/^ *^ep^* mice have increased liver lipid accumulation. (a) Liver sections from *Mrps12* ^+/+^, *Mrps12^ep^* ^/^ *^ep^* and *Mrps12^ha^* ^/^ *^ha^* mice fed a high‐fat diet were cut to 10 µm thickness and heart sections (b) were cut to 5 µm thickness, then stained with haematoxylin and eosin (H&E). Each image is representative of sections from six mice per genotype. (c) Parameters for *Mrps12* ^+/+^ *n* = 5 and *Mrps12^ha^* ^/^ *^ha^* *n* = 5 mice fed a HFD. LVEDD, left ventricular end‐diastolic diameter; LVESD, left ventricular end‐systolic diameter; FS, fractional shortening; LVDPW, left ventricular posterior wall in diastole; LVSPW, left ventricular posterior wall in systole; IVDS, intraventricular septum in diastole; IVSS, intraventricular septum in systole; HR, heart rate. Values are means ± SEM. *p < 0.05 compared with *Mrps12* ^+/+^, Student's *t* test. (d) Representative raw electrocardiographic recordings from at least six control and six *Mrps12^ha^* ^/^ *^ha^* mice fed an NCD at 6 weeks and fed a high‐fat diet at 20 weeks. Characteristic altered repolarisation in hearts of *Mrps12^ha^* ^/^ *^ha^* mice was identified as an increase in the T/ST‐wave area. (e) Electrocardiography recordings showed significant differences in the T/ST‐wave area of the *Mrps12^ha^* ^/^ *^ha^* compared to *Mrps12* ^+/+^ mice fed either a normal diet at six weeks of age or following a high‐fat diet by 20 weeks of age. Values are means ± SEM. *p < 0.05 compared with *Mrps12* ^+/+^, Student's *t* test

Recently, we showed a liver‐specific response to error‐prone mitochondrial translation and a heart‐specific defect in response to hyper‐accurate protein synthesis under a normal dietary regime (Ferreira et al., 2019). Therefore, we investigated the physiological consequences of a high‐fat diet on the liver and heart in the mutant and control mice. In contrast to the beneficial effects of the error‐prone mutation for liver function on an NCD (Ferreira et al., 2019), the *Mrps12^ep^* ^/^ *^ep^* mice fed a high‐fat diet had increased accumulation of lipid droplets in the liver compared to the control and *Mrps12^ha^* ^/^ *^ha^* mice (Figure 2a). The livers of the *Mrps12^ha^* ^/^ *^ha^* mice were most resistant to lipid accumulation, with fewer fat deposits compared to the control mice, indicating that the hyper‐accurate mutation has a protective effect in the liver (Figure 2a). These findings are consistent with increased levels of alanine aminotransferase (ALT) in the serum of the *Mrps12^ep^* ^/^ *^ep^* mice, but not control or *Mrps12^ha^* ^/^ *^ha^* mice (Figure 2a).

The HFD did not affect the heart morphology of the *Mrps12^ep^* ^/^ *^ep^* mice (Figure 2b), consistent with previous reports for these mice when they were fed an NCD (Ferreira et al., 2019). However, cardiomyocyte disarray and necrotic foci were present in the *Mrps12^ha^* ^/^ *^ha^* mice on an HFD (Figure 2b) and echocardiography identified significant changes including: increased fractional shortening, increased intraventricular septum thickness and decreased left ventricular diameter (Figure 2c), consistent with the development of hypertrophic cardiomyopathy. Histology analyses of the hearts from *Mrps12^ha^* ^/^ *^ha^* mice revealed changes that were also consistent with hypertrophy (Figure S2). Therefore, we performed electrocardiography and identified hyperacute T waves defined by increases in T‐wave area in the hearts of the *Mrps12^ha^* ^/^ *^ha^* mice, suggesting altered repolarisation through the hypertrophic myocardium (Figure 2d,e and Figure S3). The cardiac phenotype was consistent with an elevated immune response, possibly from necrotic cardiomyocytes generating inflammatory responses, that was identified in the blood profiles of the HFD fed *Mrps12^ha^* ^/^ *^ha^* mice (Figure 1d). Electrocardiography revealed that a cardiac defect was present in the mutant mice from 6 weeks of age, however, the HFD resulted in hypertrophic cardiomyopathy in the 20‐week‐old HFD fed *Mrps12^ha^* ^/^ *^ha^* mice.

### 2.3 The hyper‐accurate mutation protects from the effects of a high‐fat diet

**FIGURE 3:**
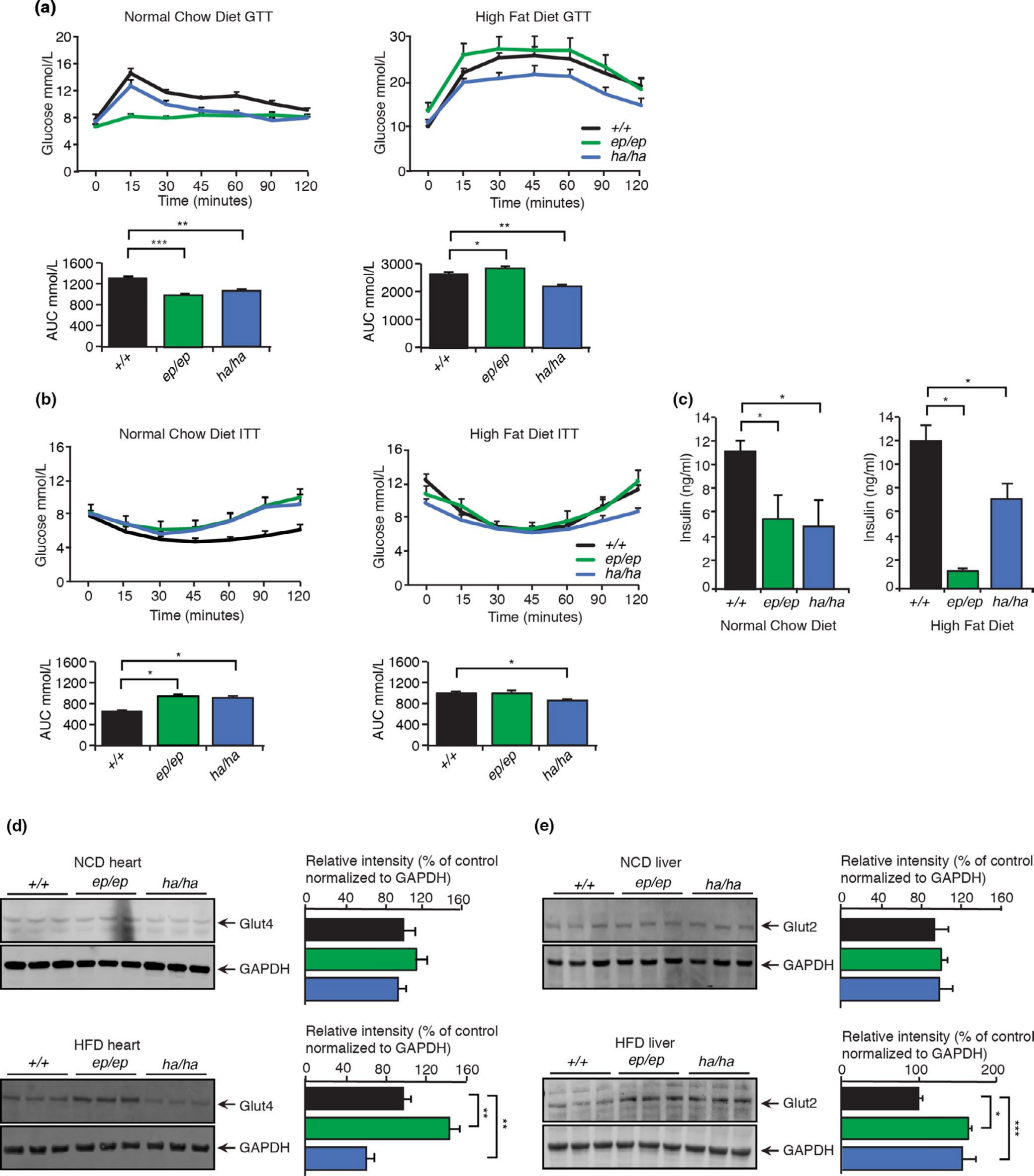
Increased translational accuracy protects from metabolic dysfunction. (a) Glucose tolerance was measured in *Mrps12^ep^* ^/^ *^ep^* (*n* = 6) and *Mrps12^ha^* ^/^ *^ha^* (*n* = 6) mice fed normal chow or high‐fat diet after 12 weeks. Quantitative values are the area under the curve (AUC) ± SEM. *p < 0.05, **p < 0.01, ***p < 0.001, Student's *t* test. (b) Insulin tolerance was measured in *Mrps12^ep^* ^/^ *^ep^* (*n* = 6) and *Mrps12^ha^* ^/^ *^ha^* (*n* = 6) mice fed a normal chow or high‐fat diet after 12 weeks. Quantitative values are the area under the curve (AUC) ± SEM. *p < 0.05, **p < 0.01, ***p < 0.001, Student's *t* test. (c) Insulin was measured in serum of *Mrps12^ep^* ^/^ *^ep^* (*n* = 5) and *Mrps12^ha^* ^/^ *^ha^* (*n* = 5) mice fed a normal and high‐fat diet. Error bars indicate SEM. *p < 0.05, Student's *t* test. Abundance of glucose transporters were examined by immunoblotting against Glut4 in hearts of *Mrps12* ^+/+^, *Mrps12^ep^* ^/^ *^ep^* and *Mrps12^ha^* ^/^ *^ha^* mice (d) and Glut2 in livers of *Mrps12* ^+/+^, *Mrps12^ep^* ^/^ *^ep^* and *Mrps12^ha^* ^/^ *^ha^* mice fed a normal or high‐fat diet (e). GAPDH was used as a loading control and the relative abundance of the proteins was analysed relative to the loading control. Images are representative of blots from *n* = 6 of each genotype. Values are means ± SEM. *p < 0.05, **p < 0.01, ***p < 0.001 Student's *t* test

To investigate the metabolic changes that may have a protective role from high caloric food intake we carried out glucose and insulin tolerance tests on *Mrps12^ep^* ^/^ *^ep^*, *Mrps12^ha^* ^/^ *^ha^* and control mice fed either an NCD or HFD. The *Mrps12^ep^* ^/^ *^ep^* and *Mrps12^ha^* ^/^ *^ha^* mice fed an NCD were more glucose tolerant compared to their control littermates (Figure 3a), suggesting that the mutations may confer a metabolic advantage. However, on the HFD, the *Mrps12^ep^* ^/^ *^ep^* mice became more glucose intolerant compared to control and *Mrps12^ha^* ^/^ *^ha^* mice, consistent with the increased lipid accumulation in the livers of the *Mrps12^ep^* ^/^ *^ep^* mice. In contrast, the *Mrps12^ha^* ^/^ *^ha^* mice were more glucose tolerant (Figure 3a). The glucose tolerance of the *Mrps12^ha^* ^/^ *^ha^* mice on both diets was consistent with their leaner phenotype and reduced lipid accumulation in their livers compared to both control and *Mrps12^ep^* ^/^ *^ep^* littermates (Figures 1b and 2a).

The insulin tolerance of the *Mrps12^ep^* ^/^ *^ep^* and *Mrps12^ha^* ^/^ *^ha^* mice was the same as that of the control mice fed an NCD (Figure 3b), consistent with their comparable weights (Figure 1a). However, on the HFD, the *Mrps12^ha^* ^/^ *^ha^* mice were insulin sensitive (Figure 3b), consistent with their increased glucose tolerance and reduced weight compared to the *Mrps12^ep^* ^/^ *^ep^* and control mice (Figures 3a and 1b). Circulating insulin levels were reduced in both *Mrps12^ep^* ^/^ *^ep^* and *Mrps12^ha^* ^/^ *^ha^* mice on both diets when compared to control mice (Figure 3c), however, the reduction in circulating insulin levels was greater in the *Mrps12^ep^* ^/^ *^ep^* mice fed on an HFD, consistent with their glucose intolerance. This suggests that the error‐prone mutation on an HFD results in reduced insulin production that contributes to glucose intolerance and lipid accumulation. In contrast, the hyper‐accurate mutation has a greater effect than the HFD and can protect the liver from increased lipid accumulation. Although the error‐prone mutation can be beneficial on an NCD, these effects are overcome by the high‐fat diet.

We analysed the levels of the glucose transporters, GLUT4 in the hearts, and GLUT2 in the livers of the mice, to determine if their expression was changed in response to the two different diets. GLUT4 levels were not significantly altered in the hearts of the mutant mice on an NCD, however, they were reduced in the *Mrps12^ha^* ^/^ *^ha^* mice compared to the control mice levels on the HFD, limiting glucose availability in the heart that may contribute to the development of hypertrophy (Figure 3d). The levels of the glucose transporter GLUT2 in the liver were similar in all of the mouse lines fed an NCD (Figure 3e). However, the HFD resulted in increased levels of GLUT2 (Figure 3e), suggesting that it may be a compensatory attempt to recover from the metabolic perturbation.

### 2.4 High caloric diet increases mitochondrial translation in *Mrps12^ep^* ^/^ *^ep^* and *Mrps12^ha^* ^/^ *^ha^* mice

**FIGURE 4:**
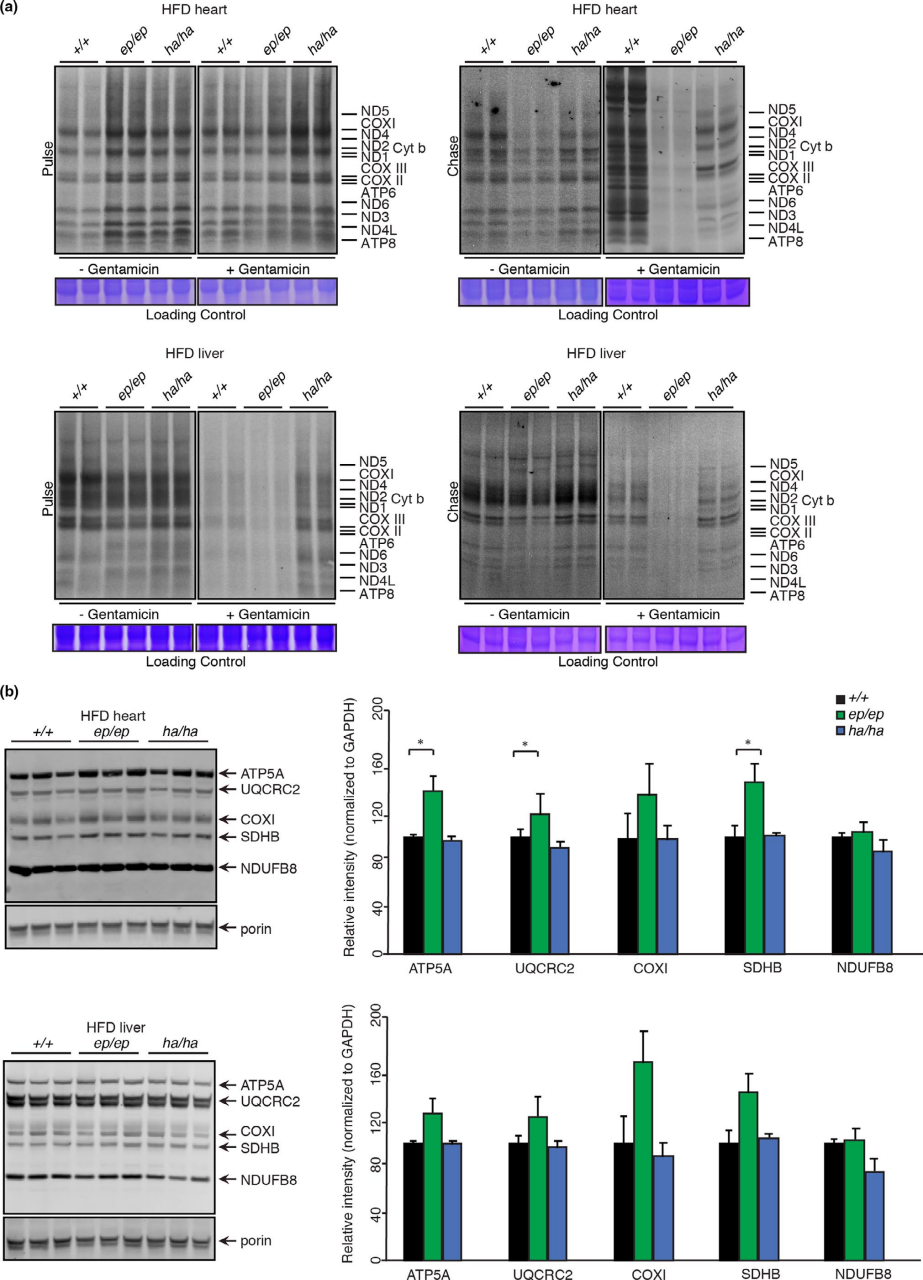
Metabolic stress causes tissue‐specific changes in translational fidelity. (a) De novo protein synthesis in heart and liver mitochondria isolated from 20‐week‐old *Mrps12* ^+/+^, *Mrps12^ep^* ^/^ *^ep^* and *Mrps12^ha^* ^/^ *^ha^* mice fed a HFD, was measured by pulse and chase incorporation of ^35^S‐labelled methionine and cysteine in the presence and absence of 0.5 µM gentamicin. Mitochondrial protein lysates were separated by SDS‐PAGE, stained with Coomassie Brilliant Blue to show equal loading and visualised by autoradiography. The gels are representative of at least three independent biological experiments using at least six mice per genotype. (b) Steady state levels of heart or liver mitochondrial proteins isolated from 20‐week‐old *Mrps12* ^+/+^, *Mrps12^ep^* ^/^ *^ep^* and *Mrps12^ha^* ^/^ *^ha^* mice fed an HFD, were analysed by immunoblotting against an OXPHOS cocktail antibody. Porin was used as a loading control. Relative abundance of proteins was analysed relative to the loading control. Images are representative of blots from *n *= 6 of each genotype. Values are means ± SEM. *p < 0.05, Student's *t* test

We measured *de* *novo* mitochondrial protein synthesis and protein stability by pulse and chase labelling to identify the effects of the mutations on a HFD (Figure 4a). Pulse labelling identified that the rate of protein synthesis was increased in heart mitochondria from the *Mrps12^ep^* ^/^ *^ep^* mice compared to control mice (Figure 4a, top left pulse panel). However, chase labelling revealed that the stability of the newly synthesised proteins was dramatically reduced compared to controls (Figure 4a, top right chase panel). This is in stark contrast to the effects of the NCD that caused a reduction of protein synthesis in the *Mrps12^ep^* ^/^ *^ep^* mice (Ferreira et al., 2019), but is consistent with *E*.*coli* and yeast mitochondria where the error‐prone mutation causes increased rates of protein synthesis (Agarwal et al., 2011; Ferreira et al., 2019). The reduced stability of the newly synthesized proteins in the error‐prone mutant mice is most likely a consequence of amino acid misincorporation causing misfolding. To determine if increased amino acid misincorporation results in destabilisation of *de* *novo* produced mitochondrial proteins we used the aminoglycoside antibiotic gentamicin. We used gentamicin because aminoglycosides have been shown to increase the sensitivity of error‐prone mutations by disrupting codon recognition and proofreading systems where accelerating domain closure results in the incorrect insertion of non‐cognate tRNAs into the peptidyl‐transferase centre (Agarwal et al., 2011). In the presence of gentamicin, the error‐prone mutation no longer increased the rate of protein synthesis during pulse labelling (Figure 4a, top left chase panel) and de novo proteins were even less stable in the chase labelling compared to their respective controls (Figure 4a, top right chase panel). These data indicate that gentamicin increases the rate of amino acid misincorporation, exacerbating protein instability and degradation caused by the MRPS12 error‐prone mutation.

Pulse labelling revealed that the hyper‐accurate mutation also increased the rate of translation compared to control mice, albeit not to the same level as the error‐prone mutation (Figure 4a top left pulse panel). In contrast to the error‐prone mutation, *de* *novo* proteins generated in *Mrps12^ha^* ^/^ *^ha^* mice were stable and comparable to the control mice (Figure 4a top right chase panel). The presence of gentamicin increased the rate of protein synthesis in the hyper‐accurate mice compared to control and error‐prone mice, and the stability of the de novo proteins was not reduced as in the error‐prone mice (Figure 4a). These results indicate that in the heart the HFD combined with the error‐prone mutation increased the rate of protein synthesis that results in the misincorporation of amino acids and reduced the stability of mitochondrially encoded proteins. Therefore an HFD stimulates a higher rate of protein synthesis in the mice with the hyper‐accurate mutation in contrast to the reduced rate of translation in these mice on an NCD (Ferreira et al., 2019) and maintains the stability of the newly produced proteins.

In the liver, the effects of the error‐prone mutation combined with an HFD caused a reduction in protein synthesis compared to controls determined by pulse labelling (Figure 4a lower left panel). Amino acid misincorporation resulted in decreased de novo protein stability, identified by chase labelling (Figure 4a lower right panel). Gentamicin had similar effects in the livers of the HFD fed error‐prone mice, where it reduced both the rate of translation and the stability of the newly synthesized proteins, by exacerbating amino acid misincorporation (Figure 4a lower panels). In the livers of the hyper‐accurate mutant mice, the rate of translation and stability of de novo proteins was not affected compared to controls (Figure 4a lower panels). These findings validate that the rate of translation is more important than its accuracy for maintaining normal heart function, which may protect from the effects of an HFD. However, in the liver the HFD compromised the rate of translation as a result of the error‐prone mutation and affected liver function, causing increased lipid accumulation and glucose resistance.

We used immunoblotting to investigate the net effects on mitochondrial OXPHOS protein levels as a result of altered translation rates and protein stability on an HFD in the mutant mice compared to controls (Figure 4b). Despite decreased de novo protein stability, the steady‐state levels of OXPHOS proteins in the *Mrps12^ep^* ^/^ *^ep^* mice fed an HFD were increased and unchanged in heart and liver mitochondria, respectively. In heart and liver mitochondria isolated from *Mrps12^ha^* ^/^ *^ha^* mice, the HFD did not change the steady‐state levels of the OXPHOS proteins and they were comparable to control mice (Figure 4b). This suggests that the pathological changes in the hearts of the *Mrps12^ha^* ^/^ *^ha^* mice and the lipid accumulation in the livers of the *Mrps12^ep^* ^/^ *^ep^* mice was not a consequence of OXPHOS dysfunction, but instead the error‐prone and hyper‐accurate mutations have an independent and tissue‐specific effect on a high‐calorie diet.

### 2.5 High‐fat diet and hyper‐accurate mitochondrial protein synthesis result in increased levels of mitochondrial ribosomal proteins in the heart

Previously we have shown that neither of the mutations in mice fed an NCD affected the abundance of mitoribosomal proteins (Ferreira et al., 2019). Here we found that the hyper‐accurate mutation caused an increase in mitoribosomal proteins in the hearts of mice fed an HFD, compared to control and error‐prone mutant mice (Figure S4a). The error‐prone mutation caused a minor reduction in specific mitoribosomal proteins in the heart but not the liver compared to control mice. We used sucrose gradient fractionation to resolve the mitochondrial subunits and translating mitoribosomes followed by immunoblotting for markers of the small and large ribosomal subunits to show that neither of the mutations altered the biogenesis and engagement of the mitoribosomes on an HFD (Figure S4b). These findings indicate that altered de novo translation rates in the mutant mice (Figure 4a) were a direct consequence of the error‐prone and hyper‐accurate mutation in the MRPS12 protein that affects the opening of the decoding site (Agarwal et al., 2011; Ferreira et al., 2019).

**FIGURE 5:**
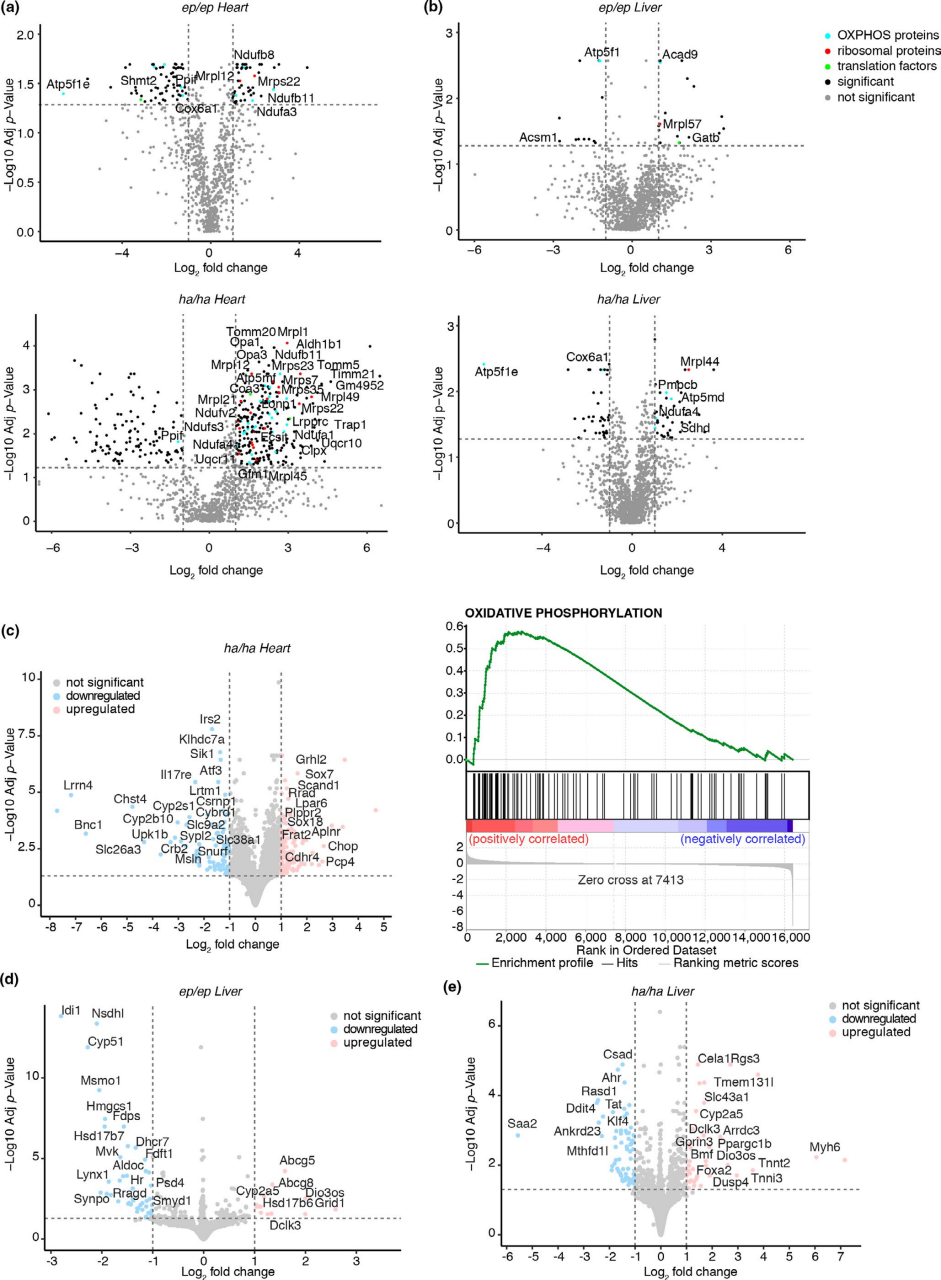
Proteomic and transcriptomic consequences of altered translational fidelity during metabolic stress. Quantitative proteomic analysis of heart (a) or liver (b) mitochondrial proteins from 20‐week‐old *Mrps12^ep^* ^/^ *^ep^* and *Mrps12^ha^* ^/^ *^ha^* mice fed a HFD relative to their respective controls on a normal diet. Transcriptome‐wide RNA‐seq was carried out on total heart or liver RNA from 20‐week‐old *Mrps12* ^+/+^, *Mrps12^ep^* ^/^ *^ep^* and *Mrps12^ha^* ^/^ *^ha^* mice fed a HFD, and differential expression analyses were performed. Volcano plots show the significant differences (*p* < 0.05) in positively and negatively correlated transcripts relative to controls. Transcriptome‐wide changes in hearts from 20‐week‐old a *Mrps12^ha^* ^/^ *^ha^* compared to *Mrps12* ^+/+^ mice (c) and the most significantly changing gene ontologies are shown for oxidative phosphorylation. The most significantly changing transcripts in the livers of *Mrp12^ep^* ^/^ *^ep^* and *Mrps12^ha^* ^/^ *^ha^* mice are shown in (d) and (e), respectively

Proteomic analyses of mitochondria from the mutant mice on an HFD were consistent with the immunoblotting results: the hyper‐accurate mutation in the heart caused an increase in mitoribosomal proteins (Figure 5a and Figure S5a), and the error‐prone mutation had a minor effect on the mitoribosomal proteins (Figure 5a). The effects on mitoribosomal protein levels in the livers of the mutant mice fed an HFD were negligible, consistent with the immunoblotting results (Figure 5b). In addition, OXPHOS proteins were increased in the hyper‐accurate mutant mice fed an HFD (Figure 5a), consistent with the increased rate of de novo protein synthesis. Proteomic analysis of isolated mitochondria from livers of the mutant mice on an HFD revealed minimal to no effects on the steady‐state levels of OXPHOS proteins (Figure 5b), consistent with the immunoblotting results. In the hearts of the *Mrps12^ep^* ^/^ *^ep^* the mitochondrially encoded ATP8 protein was reduced relative to nuclear‐encoded OXPHOS proteins, validating the de novo translation results that the stability of mtDNA encoded proteins is reduced (Figure S5b). Their reduced stability may be a consequence of increased levels of the protease AFG3L2 in the hearts of these mice (Figure S5c), possibly in an effort to remove mistranslated proteins. Interestingly, the proteomic analyses identified increased levels of heart hypertrophy markers, such as aldehyde dehydrogenase (Aldh1b1; Chi et al., 2006) and glycine N‐acyltransferase‐like protein (Gm4952; Bell et al., 2011; Rardin et al., 2013), in the hearts of the *Mrps12^ha^* ^/^ *^ha^* mice (Figure 5a), consistent with increased autophagy (Figure S5d) and hypertrophy identified in their hearts. The increased levels of acetyl coenzyme A synthetase (Acsm1) that is important for lipoic acid utilization and increased glucose uptake, may help protect the livers of the *Mrps12^ha^* ^/^ *^ha^* mice (Figure 5b) from lipid accumulation on the HFD. In contrast, Acsm1 is reduced in the liver mitoproteomes of *Mrps12^ep^* ^/^ *^ep^* mice that have increased lipid accumulation, as well as proteins involved in fatty acid oxidation such as Acad9. The glutamine tRNA aminotransferase, Gatb, is also reduced in the *Mrps12^ep^* ^/^ *^ep^* mice, likely as a compensatory response to amino acid misincorporation (Figure 5b).

### 2.6 Cellular responses to altered mitochondrial translational fidelity are tissue‐specific

The hyper‐accurate mutation had the most profound effect on heart function and this was further evident from the increased levels of mitochondrial biogenesis proteins involved in protein import (Tomm20, Timm21, Tomm5, Timm50, Timm10b), dynamics (Opa1, Opa3) and proteostasis (Clpx, Trap1 and Lonp1) in our proteomics analyses. The increase in these factors can often be an indicator of mitochondrial stress and therefore we investigated the transcriptional changes caused by the hyper‐accurate mutant mice on an HFD. Differential expression analyses for *Mrps12^ha^* ^/^ *^ha^* mouse hearts revealed increases in transcriptional stress response genes, including *Chop* and cardiomyocyte transcription factors (*Pcp4*, *Sox18*) that regulate cardiac tone and contraction (Figure 5c) in an effort to combat blood flow defects. The OXPHOS genes were also elevated (Figure 5c), consistent with the proteomic analyses. There was a reduction in solute carriers and transporters including the sodium‐coupled amino acid transporter *Slc38a1* and cytochrome p450 transcripts, consistent with cardiac defects in these mice. There were no significant changes identified in the hearts of the *Mrps12^ep^* ^/^ *^ep^* mice on an HFD indicating that amino acid misincorporation is better tolerated in the hearts since there were no cardiac defects as a result of the error‐prone mutation. In contrast, the error‐prone mutation in the liver caused changes in the expression of genes involved in steroid metabolism (Figure 5d), in response to the elevated fat accumulation in the livers of these mice. The hyper‐accurate mutation appears to protect the liver from lipid accumulation by upregulation of hepatic stress response genes, such as *Foxa2*, the amino acid transporter *Slc43a1* and the cytochrome P450 *Cyp2a5* that can alleviate the stress induced by the HFD (Figure 5e).

We examined mTOR signalling, because of the stress responses identified in the *Mrps12^ha^* ^/^ *^ha^* mice, by immunoblotting to measure the level of S6 and 4E‐BP phosphorylation. Although S6 phosphorylation was not altered in the hearts or livers of *Mrps12^ha^* ^/^ *^ha^* mice, in the *Mrps12^ep^* ^/^ *^ep^* mice the steady‐state levels of S6 and S6 phosphorylation were significantly reduced in both tissues (Figure S6a,b). A similar trend was found for the steady‐state and phosphorylated levels of 4E‐BP (Figure S6a,b). Next, we analysed the effects of the mutation in both tissues on Akt, which is a component of the PI3K (phosphatidylinositol 3‐kinase) pathway that can be an upstream positive regulator of S6 activity via mTOR (Hay & Sonenberg, 2004). The steady‐state levels of Akt were not different in the hearts of the mutant mice compared to controls (Figure S6a,b), however, Akt phosphorylation was significantly decreased in the livers of *Mrps12^ep^* ^/^ *^ep^* mutant mice (Figure S6b) but not in the livers of *Mrps12^ha^* ^/^ *^ha^* mice. It is possible that this reduction in Akt signalling may predispose the livers of the *Mrps12^ep^* ^/^ *^ep^* mutant mice to increased lipid accumulation. The AMP‐activated protein kinase (AMPK) which is regulated by changes in intracellular ATP levels and activated by phosphorylation was not affected by the mutations or HFD in either tissue (Figure S6a,b), indicating that ATP levels are not changing significantly to affect its activity. However, although unaltered on an NCD (Figure S6c), the phosphorylation of the transcriptional co‐activator Yes‐associated protein 1 (YAP) that controls cell proliferation and suppresses apoptosis was reduced in the livers of the *Mrps12^ep^* ^/^ *^ep^* mutant mice on an HFD (Figure S6d). This indicates that in the *Mrps12^ep^* ^/^ *^ep^* mutant mice reduced signalling via Akt and YAP phosphorylation may contribute to the lipid accumulation in the liver whereas hyper‐accurate mutant mice were protected from liver dysfunction as these signalling pathways were not affected.

Amino acids regulate and provide the building blocks for protein synthesis, therefore we investigated the effects of the error‐prone and hyper‐accurate mutation on amino acid content in the hearts and livers of the mutant mice compared to controls by mass spectrometry. We found the most significant changes in total amino acid levels in the hearts of the hyper‐accurate mutant mice (Figure S7a), consistent with a reduction in the expression of amino acid transporters identified by RNA‐seq. The levels of 4‐hydroxyproline and α‐aminobutyric acid were increased in the hyper‐accurate mice, which was consistent with the cardiomyopathy that was identified in these mice as these amino acids are predictive biomarkers of heart disease. Homoserine, serine and methionine were also elevated in the hearts of the hyper‐accurate mutants, however, the branched‐chain amino acids, leucine, isoleucine and valine were all reduced (Figure S7a). In contrast to the heart, in the liver, only the error‐prone mutation affected the levels of specific amino acids (Figure S7b), consistent with the observed liver dysfunction and absence of heart dysfunction observed in these mice. Citrulline and ornithine were reduced in the livers of the error‐prone mutant mice, suggesting changes in the citric acid cycle, likely due to the increased lipid accumulation in the livers. In addition, methionine, glycine and tyrosine were reduced, possibly a consequence of reduced citric acid cycle function. Next, we measured amino acid levels in heart and liver mitochondria from the control and mutant mice fed an HFD. In heart mitochondria from the hyper‐accurate mutant mice the essential and conditionally essential amino acid levels of methionine, threonine and valine as well as, proline and serine, respectively, were significantly reduced (Figure S7c), suggesting that their import, use or biosynthesis within mitochondria may be impaired. The reduction of mitochondrial levels of methionine and serine (Figure S7c), required for one‐carbon metabolism, may activate the stress response via transcription factors such as CHOP in an attempt to stimulate their increased uptake and biosynthesis in the cytoplasm (Figure S7a). In contrast, in the heart of the error‐prone mutant mice, mitochondrial glycine and sarcosine levels were significantly increased which may confer protective effects by supporting carbon one metabolism, thereby compensating for the reduction in methionine levels. In liver mitochondria from the error‐prone mutant mice, sarcosine levels were specifically reduced compared to both control and hyper‐accurate mice (Figure S7d), likely as a result of reduced glycine levels in the liver (Figure S7b). The reduction in amine intermediate metabolites in the livers of the error‐prone mice (1‐methylhistidine, 3‐methylhistidine, 4‐hydroxyproline, aminoadipic acid, α‐aminoisobutyric acid) indicated impaired amino acid import and metabolism within mitochondria. There were no changes in mitochondrial amino acid levels in the livers of the hyper‐accurate mutant mice consistent with the absence of changes in total amino acid levels and the absence of liver dysfunction. These data suggest that changes in the fidelity of translation have pronounced effects on amino acid content that could impact the pathologies of each of the tissues, as opposed to OXPHOS defects that are not significantly apparent in these mutant mice when they were fed a high‐fat diet.

### 2.7 The rate of translation is more important for lifespan in mice and worms

**FIGURE 6:**
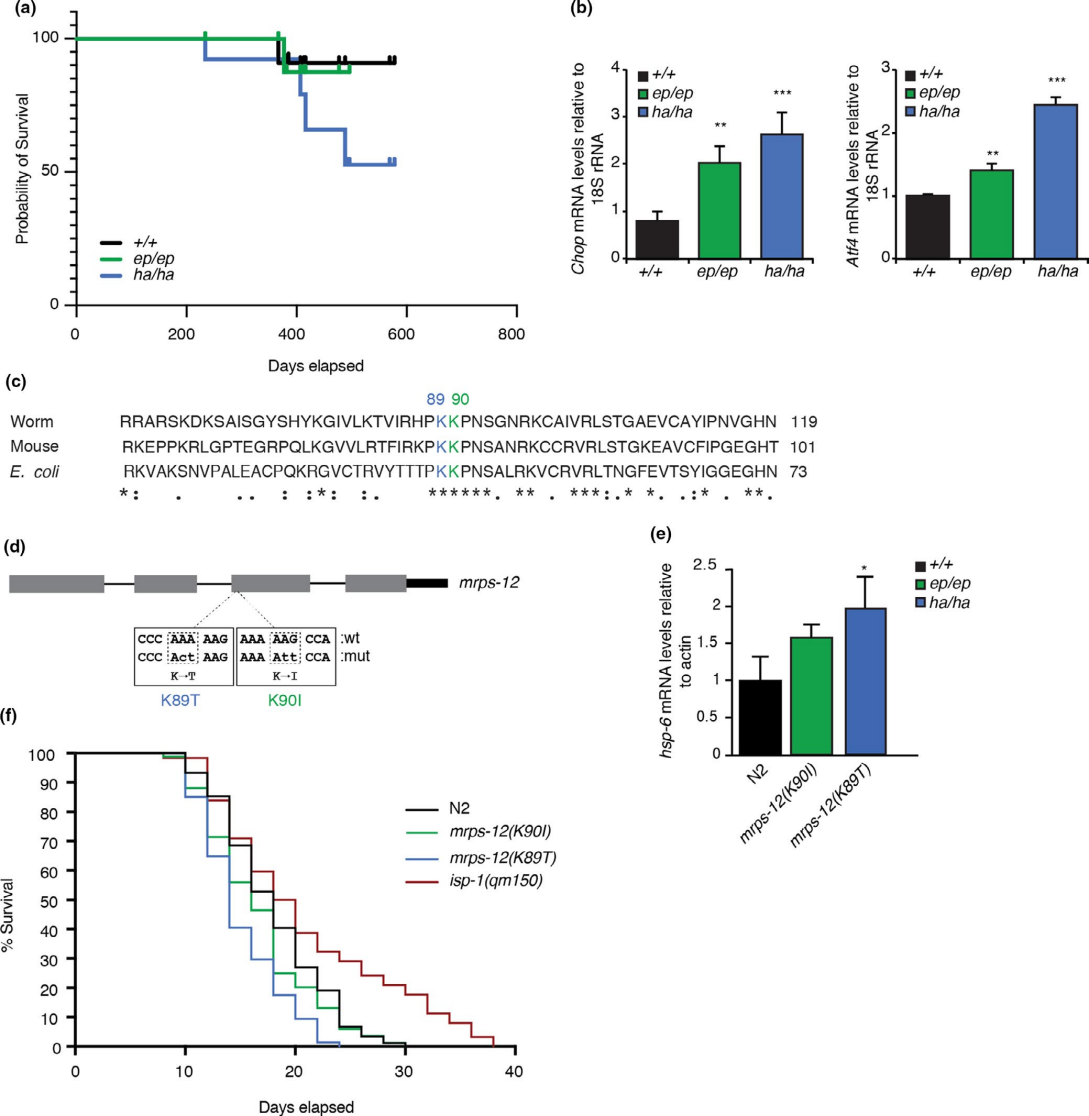
The rate of translation protects the life spans of mammals and worms. (a) The lifespan of *Mrps12* ^+/+^, *Mrps12^ep^* ^/^ *^ep^* and *Mrps12^ha^* ^/^ *^ha^* mice fed a NCD was measured over 2 years, where the *Mrps12^ha^* ^/^ *^ha^* mice have a reduced lifespan. (b) Relative expression of *Chop* and *Atf4* in *Mrps12* ^+/+^, *Mrps12^ep^* ^/^ *^ep^* and *Mrps12^ha^* ^/^ *^ha^* aged mice fed a NCD, determined by qRT‐PCR and normalized to 18S rRNA. Values are means ± SEM. **p < 0.01, ***p < 0.001 compared with *Mrps12* ^+/+^ using a Student's *t* test. (c) The error‐prone and hyper‐accurate mutations are conserved from bacteria to worms and mammals, shown by the primary protein sequence alignment. The error‐prone mutation is shown in green and the hyper‐accurate mutation is shown in blue. (d) Schematic of the *Mrps12* gene in *Caenorhabditis elegans* with the error‐prone and hyper‐accurate mutations are introduced converting lysine to threonine and isoleucine at positions 89 and 90, respectively. (e) Relative expression of *hsp*‐*6* in *mrps*‐*12* ^+/+^ (N2, wild type), *mrps*‐*12^ep^* ^/^ *^ep^* and *mrps*‐*12^ha^* ^/^ *^ha^* *C*. *elegans*, determined by qRT‐PCR, as a measure of the mitochondrial unfolded protein response. (f) The life span of *mrps*‐*12* ^+/+^, *mrps*‐*12^ep^* ^/^ *^ep^*, *mrps*‐*12^ha^* ^/^ *^ha^* and *isp*‐*1(qm150)* *C*. *elegans* was measured over 40 days and the % survival is shown. The *isp*‐*1(qm150)* mutation is included as a positive control for a mutation known to extend life span in worms

An error‐prone mutation (P50R) in the MRPS12 protein shortened the life span in yeast, while the hyper‐accurate K71T mutation extended yeast life span (Suhm et al., 2018). Here we investigated the effects of the error‐prone and hyper‐accurate mutation on life span in mice fed an NCD. We found that the life span of the error‐prone mutant mice was similar to control mice (Figure 6a), however, the hyper‐accurate mutation significantly reduced the lifespan of mice that died or had to be euthanized as a result of cardiac failure (Figure 6a). The hyper‐accurate mutation resulted in increased expression of the stress response genes *Chop* and *Atf4* that are associated with cardiac dysfunction and consequently reduced lifespan (Figure 6b). Although for ethical reasons we could not examine the life span of the mutant mice on an HFD, we conclude that the rate of translation is more important for health than the fidelity of protein synthesis, consistent with our previous findings (Ferreira et al., 2019). We also evaluated the effects of the conserved mutations in the *Mrps12* gene (Figure 6c) that we introduced in *C*. *elegans* (Figure 6d) at positions K89T and K90I (equivalent to K71T and K72I in mice, respectively) to test the effects of error‐prone or hyper‐accurate translation in the worm. The increased levels of *hsp*‐*6* in the hyper‐accurate mutant worms indicated an increased mitochondrial unfolded protein response (Figure 6e) and this resulted in a reduced life span of these mutants compared to control worms (Figure 6f). The error‐prone mutation had a similar life span to the control worms (Figure 6f), further indicating that in animals the rate of translation is important for survival.

## 3 DISCUSSION

Error‐prone and hyper‐accurate mutations in the mitoribosome have opposing physiological effects in yeast and mammals; in yeast, the error‐prone mutation results in an increased rate of translation that elicits general transcriptional stress resulting in reduced lifespan (Suhm et al., 2018). In mammals, the same mutation reduces the rate of translation on an NCD that is beneficial for liver function in mice (Ferreira et al., 2019). This was because the low‐level errors in proteins induced a specific transcriptional stress response that increased mitochondrial biogenesis (Ferreira et al., 2019). Although error‐prone translation causes a general stress response in bacteria and yeast, mammals can cope until additional stress, such as the high‐fat diet, is imposed and adverse effects are elicited.

In mammals, there is a tissue‐specific regulation of mitochondrial translation in response to increased calorie intake, where the error‐prone mutation resulted in increased lipid accumulation in the liver, but heart function was not affected. The increased rate of translation in the hearts of the error‐prone mutant mice compensated for the reduced stability of mitochondrial proteins. The increased rate of protein synthesis ensured the steady‐state levels of mitochondrial OXPHOS proteins were not reduced. In contrast, the hyper‐accurate mutation resulted in a leaner phenotype that protected from metabolic stress and lipid accumulation in the liver but caused hypertrophic cardiomyopathy. These findings reflect the complex signalling pathways that differ between post‐mitotic and highly proliferative tissues such as the heart and liver, respectively.

The error‐prone K72I mutation did not affect the life span of worms and mice, however, another error‐prone P50R mutation in MRPS12 reduced the viability of yeast (Suhm et al., 2018). It is important to note that the K72I mutation in MPRS12 did not affect the viability of yeast on non‐fermentable carbon sources (Suhm et al., 2018), suggesting that the position of the mutation within MRPS12 could play a role in the severity of the yeast phenotype. This is supported by the finding that the K72I mutation in MPRS12 confers a regenerative advantage in the livers of mice fed on a normal diet by activating a transcriptional stress response that increases mitochondrial biogenesis (Melber & Haynes, 2018; Molenaars et al., 2020; Rudler et al., 2020). Furthermore, a V338Y mutation in another mitoribosomal protein, MRPS5, required for translational fidelity, that led to increased mitochondrial mistranslation had adverse effects, causing enhanced anxiety and susceptibility to noise‐induced hearing loss (Akbergenov et al., 2018). Therefore, functional consequences of mitochondrial mistranslation likely depend on the nature of the mutations in ribosomal proteins that mediate tRNA recognition and mRNA proofreading.

The hyper‐accurate K71T mutation in MRPS12 is conserved between yeast, worms and mice and resulted in opposing effects between yeast and animals. Although this mutation extended the yeast lifespan by increasing the proteostatic capacity of the cells (Suhm et al., 2018), the same mutation shortened the lifespan of worms and mice where it caused early onset cardiomyopathy. The different effects and consequences from the altered mitochondrial protein fidelity between yeast and animals reflect the evolutionary tissue specification that can adapt and respond via specialised regulatory responses. This is evident from the differences in transcriptional and proteostatic changes between yeast, worms and mice and response to stresses such as oxidative damage (Ferreira et al., 2019; Suhm et al., 2018) or HFD. In worms, the error‐prone and hyper‐accurate mutations upregulated the mitochondrial unfolded protein response (mtUPR), likely in an effort to restore homeostasis during mitochondrial stress (Melber & Haynes, 2018), albeit the response was greater for the hyper‐accurate mutation consistent with the reduced lifespan of these worms. This indicates that the induction of the mtUPR in worms might be more sensitive than in mammals in detecting mitochondrial dysfunction. Nevertheless, the fact that both worms and mammals can mount a stress response that is specific to mitochondria, unlike the general Msn2/4‐driven stress response in yeast, might differentiate the ability of animals to adapt to a mitochondrial mistranslation in a more sophisticated manner to compensate for its physiological effects.

In the hyper‐accurate mutant mice, the cardiac damage combined with the effects of the high‐fat diet led to a reduction in solute carriers required for cardiac contractility and tone and contributed to the development of cardiac hypertrophy. However, the upregulation of the stress responses via *Chop* and increased mitochondrial biogenesis as a compensatory measure were not able to rescue the function of the *Mrps12^ha^* ^/^ *^ha^* hearts and reduced their lifespan. In contrast, the error‐prone mutation did not affect heart function, instead, the increased rate of protein synthesis was sufficient to maintain the OXPHOS energy requirements without eliciting changes in transcriptional or cell signalling pathways. However, the error‐prone mutation combined with the HFD resulted in liver dysfunction and lipid accumulation in response to reduced levels of lipid metabolism and YAP‐mediated signalling, consistent with glucose intolerance. The tissue‐specific pathologies in response to changes in translational fidelity during metabolic stress were found to impact the levels of amino acids, where reduction in the citric acid cycle precursors in the error‐prone mutant mice was consistent with liver dysfunction. In turn, the reduction of branched‐chain amino acids in the hearts of the mutant mice suggests that β‐oxidation may be compromised and thereby contribute to the development of hypertrophic cardiomyopathy. This is in contrast to the dilated cardiomyopathy that develops in the hyper‐accurate mutant mice fed a normal diet caused by a consistently reduced rate of translation and consequent reduction in OXPHOS function (Ferreira et al., 2019). However, during metabolic stress, the high‐fat diet does not reduce OXPHOS function as the rate of translation is similar to the control mice on the same diet, but instead, the changes in the use of amino acids may instead contribute to the onset of hypertrophy in the heart. The reduction of amino acids, such as methionine and serine, in mitochondria that are required for one‐carbon metabolism likely activates the stress response, which in turn upregulates their uptake and biosynthesis in the cytoplasm. These models indicate that changes in translational fidelity can activate cellular responses via metabolites independently or prior to OXPHOS dysfunction. Also, it cannot be excluded that effects other than mistranslation might contribute to the identified phenotypes. Altered mitochondrial translation resulting in defects that precede OXPHOS deficiency has been observed before in a mouse model lacking the mitochondrial aspartyl‐tRNA synthetase that affects translation (Dogan et al., 2014).

Our study highlights that in animals the rate of translation is more important than the accuracy of protein synthesis and is required for a normal lifespan. Beyond this, the tissue‐specificity that underlies the transcriptional and cell signalling responses to mitochondrial mistranslation can confer protective or damaging effects. Therefore, future work should focus on tissue‐specific transcriptional responses that may be targeted for treatments for diseases and ageing involving defects in energy metabolism.

## 4 EXPERIMENTAL PROCEDURES

### 4.1 Animals and housing

Homozygous mutant *Mrps12* ^ep/ep^ and *Mrps12* ^ha/ha^ mice were generated as described previously (Ferreira et al., 2019) and housed in standard cages (45 cm ×29 cm ×12 cm) under a 12‐h light/dark schedule (lights on 7 am to 7 pm) in controlled environmental conditions of 22 ± 2℃ and 50 + 10% relative humidity. Male mice were fed an NCD (Rat & Mouse Chow, Specialty Foods) or a high‐fat diet (19 MJ/kg, 35% of energy from carbohydrate, 42% from fat, 23% from protein; Specialty Feeds) and water was provided ad libitum. The study were approved by the Animal Ethics Committee of the UWA and performed in accordance with Principles of Laboratory Care (NHMRC Australian code for the care and use of animals for scientific purposes, 8th Edition 2013).

### 4.2 Mitochondrial isolation

Mitochondria were collected from homogenized hearts and livers and isolated by differential centrifugation as described previously (Perks et al., 2018; Rackham et al., 2016).

### 4.3 Sucrose gradient fractionation

Sucrose gradient fractionation was carried out as described previously (Rackham et al., 2016; Richman et al., 2016).

### 4.4 Immunoblotting

Specific proteins were detected using rabbit polyclonal antibodies against: MRPL44, MRPL37, MRPL23, MRPS12, MRPS16 (Proteintech, diluted 1:1000), MRPS34 (Sigma, diluted 1:1000), GAPDH, phosphorylated and non‐phosphorylated S6, phosphorylated and non‐phosphorylated Akt, phosphorylated and non‐phosphorylated 4EBP (Cell Signalling Technology, diluted 1:500) and mouse monoclonal antibodies against: phosphorylated and non‐phosphorylated AMPKα (Cell Signalling Technology, diluted 1:500), COXII (Abcam, diluted 1:1000), phosphorylated and non‐phosphorylated YAP, AFG3L2, LC3A/B, Glut2, Glut4, and Total OXPHOS Rodent WB Antibody Cocktail (Abcam, Diluted 1:1000) in Odyssey Blocking Buffer (Li‐Cor). IR Dye 800CW Goat Anti‐Rabbit IgG or IRDye 680LT Goat Anti‐Mouse IgG (Li‐Cor) secondary antibodies were used and the immunoblots were visualized using an Odyssey Infrared Imaging System (Li‐Cor).

### 4.5 Proteomics

Proteomics was carried out on mitochondria isolated from hearts or livers from five *Mrps12* ^+/+^, *Mrps12^ep^* ^/^ *^ep^* and *Mrps12^ha^* ^/^ *^ha^* mice fed either a chow or high‐fat diet at 20 weeks using methods as described previously (Rudler et al., 2019) and as detailed in the Supporting Information. Protein abundance results were visualized with OmicsVolcano (Kuznetsova et al., 2021) and Tableau v2020.4.

### 4.6 RNA isolation and qRT‐PCR

RNA was isolated from total hearts or livers from three *Mrps12* ^+/+^, *Mrps12^ep^* ^/^ *^ep^* and *Mrps12^ha^* ^/^ *^ha^* mice fed either a chow or high‐fat diet at 20 weeks, using a bead‐beating RNA extraction strategy based on the miRNeasy Mini kit (Qiagen) and qRT‐PCR was performed as described previously (Rudler et al., 2019).

### 4.7 Transcriptomics

RNA sequencing was performed on total RNA from three *Mrps12* ^+/+^, three *Mrps12^ep^* ^/^ *^ep^* and three *Mrps12^ha^* ^/^ *^ha^* mice fed a high‐fat diet. Sequencing was performed using the Illumina HiSeq platform, according to the Illumina Tru‐Seq protocol and as we have done previously (Perks et al., 2018; Rackham et al., 2016; Siira et al., 2018) and detailed in the Supporting Information.

### 4.8 Amino acid measurements

Amino acids were extracted in 50:50 water/methanol by homogenizing 20 mg heart or liver tissue or of the isolated liver (5–7 mg) and heart (1 mg) mitochondria from five *Mrps12* ^+/+^, *Mrps12^ep^* ^/^ *^ep^* and *Mrps12^ha^* ^/^ *^ha^* mice fed a high‐fat diet, using a bead‐beater. The solvent was analysed by targeted reversed‐phase high‐speed quantitative ultra‐performance liquid chromatography‐tandem mass spectrometry (UPLC‐MS) analysis as previously described (Gray et al., 2017).

### 4.9 Analysis of peripheral blood

Differential cell counts were performed on tail vein blood using a Hemavet HV950FS blood analyser (Drew Scientific).

### 4.10 Translation assays

*In organello* translation assays were carried out in isolated heart and liver mitochondria from *Mrps12* ^+/+^, *Mrps12^ep^* ^/^ *^ep^* and *Mrps12^ha^* ^/^ *^ha^* mice fed either a chow or high‐fat diet at 20 weeks as described before (Rackham et al., 2016; Richman et al., 2016).

### 4.11 Histology

Histological analyses for hearts and livers were carried out as described previously (Ferreira et al., 2019; Richman et al., 2015) and as outlined in the Supporting Information.

### 4.12 Metabolic studies

Intraperitoneal glucose tolerance tests and insulin tolerance tests were performed on mice that were fasted for 5 h as described previously using 1 U/kg insulin for the ITT (Perks et al., 2017). Cardiac blood samples were taken to measure insulin using standard ELISA kits according to the manufacturer's instructions. Data were analysed using an online software program (www.elisaanalysis.com), and the area under the curve was calculated using the trapezoidal rule with Microsoft Excel.

### 4.13 Electrocardiography and echocardiography

The ECG (lead II) was recorded with s.c. bipolar leads for 10 min under light methoxyflurane anaesthesia. Parameters were measured on signal‐averaged complexes derived from 1 s of contiguous data (10 heartbeat cycles) using LabChart software. QT interval was corrected for heart rate using the Mitchell methods instead of Bazett's formula because it is meant for higher heart rates, as found in mice (Mitchell et al., 1998). T‐wave area measured as integral (mV·s) above baseline (Zhang et al., 2015). In parallel experiments, echocardiograms were recorded using an i13L probe on a Vivid 7 Dimension (GE Healthcare) as previously described (Ferreira et al., 2019).

### 4.14 Generation and testing of *C*. *elegans* mutants

The strain MQ887 [*isp*‐*1(qm150)*] and wild‐type (N2) were provided by the *Caenorhabditis* Genetics Centre (CGC). Standard *C*. *elegans* culture and maintenance was performed as previously described (Brenner, 1974). The generation of CRISPR‐Cas9 mutant strains is described in the Supporting Information. Lifespan assays were carried out essentially as described previously (Amrit et al., 2014) and in the Supporting Information.

### 4.15 Statistical analysis

All data shown are mean ± standard deviation or standard error of the mean (SEM) and statistical significance was calculated using a two‐tailed Student's *t* test.

## CONFLICT OF INTEREST

None declared.

## AUTHOR CONTRIBUTIONS

O.R. and A.F. conceived the project. O.R. and A.F. designed the experiments. All authors conducted and analysed the experiments. T.R. and A.F. wrote the manuscript and the other authors edited and approved the manuscript.

## Supporting information

Supplementary Material

## Data Availability

The Gene Expression Omnibus (GEO) accession number for the data reported in this paper is GSE159014. The mass spectrometry proteomics data have been deposited to the ProteomeXchange (http://proteomecentral.proteomexchange.org) via the PRIDE partner repository with the dataset identifiers PXD021762 and PXD024633. All data needed to evaluate the conclusions in the paper are present in the paper and the Supporting Information. Reasonable requests for resources should be addressed to and will be fulfilled by the corresponding author.
