# Supplementary material for: Mitochondrial mistranslation modulated by metabolic stress causes cardiovascular disease and reduced lifespan
Source: Aging Cell. 2021 Jun 7;20(7):e13408. doi: 10.1111/acel.13408 (PMC8282274; doi:10.1111/acel.13408)
Supplement: Supplementary file 1 — Supplementary Material [file ACEL-20-e13408-s001.docx]

**Supplemental Information**

**Mitochondrial mistranslation modulated by metabolic stress causes cardiovascular disease and reduced lifespan**

Tara R. Richman^1,2,3^, Judith A. Ermer^1,2,3^, Stefan Siira^1,2,3^, Irina Kuznetsova^1,2,3^, Christopher A. Brosnan^4^, Giulia Rossetti^1,2,3,5^, Jessica Baker^1,2,3,5^, Kara Perks^1,2,3,5,6^, Henrietta Cserne Szappanos^8^, Helena M. Viola^8^, Nicola Gray^9^, Mark Larance^10^, Livia C. Hool^8,11^, Steven Zuryn^4^, Oliver Rackham^1,2,3,5,6,7^ and Aleksandra Filipovska^1,2,3,5,12^*

^1^Harry Perkins Institute of Medical Research and ^2^ARC Centre of Excellence in Synthetic Biology, QEII Medical Centre, Nedlands, Western Australia 6009, Australia

^3^Centre for Medical Research, The University of Western Australia, QEII Medical Centre, Nedlands, Western Australia 6009, Australia

^4^Clem Jones Centre for Ageing Dementia Research, Queensland Brain Institute, The University of Queensland, Brisbane, Queensland, 4072 Australia

^5^Telethon Kids Institute, Northern Entrance, Perth Children's Hospital, 15 Hospital Avenue, Nedlands, Western Australia, Australia

^6^School of Pharmacy and Biomedical Sciences, Curtin University, Bentley, Western Australia 6102, Australia

^7^Curtin Health Innovation Research Institute, Curtin University, Bentley, Western Australia 6102, Australia

^8^School of Human Sciences, The University of Western Australia, 35 Stirling Highway, Nedlands, Western Australia 6009, Australia

^9^Australian National Phenome Centre, Centre for Computational and Systems Medicine, Health Futures Institute, Murdoch University, Harry Perkins Building, Perth, Westernd Australia, WA 6150, Australia

^10^Charles Perkins Centre, School of Life and Environmental Sciences, University of Sydney, Camperdown, NSW, Australia

^11^Victor Chang Cardiac Research Institute, Sydney, NSW, Australia

^12^School of Molecular Sciences, The University of Western Australia, Crawley, Western Australia 6009, Australia

* Lead contact: [aleksandra.filipovska@uwa.edu.au](mailto:aleksandra.filipovska@uwa.edu.au)

**CONTENTS**

**Supplementary Figure S1.** The effects of altered translational fidelity on food intake

**Supplementary Figure S2.** Increased accumulation of blood in the heart of *Mrps12^ha/ha^* mice

**Supplementary Figure S3.** Electrocardiographic measurements

**Supplementary Figure S4.** The stability of the mitochondrial ribosome is not affected by metabolic stress.

**Supplementary Figure S5.** The effects of altered fidelity of mitochondrial translation on mitochondrial protein stability, protease levels and autophagy.

**Supplementary Figure S6.** The effects of altered translational fidelity on protein signalling.

**Supplementary Figure S7.** Altering the fidelity of mitochondrial translation causes changes in the levels of specific amino acids under metabolic stress.

**
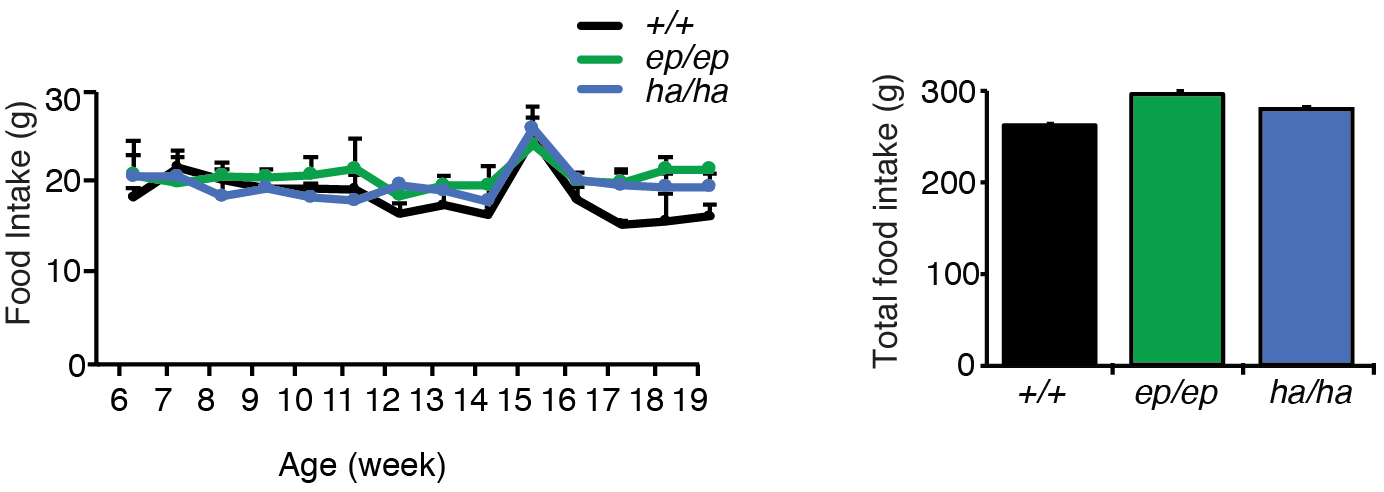
**

**Figure S1. Related to Figure 1.** The effects of altered translational fidelity on food intake. Weekly food intake during 12 weeks of HFD feeding between *Mrps12^+/+^, Mrps12^ep/ep^* and *Mrps12^ha/ha^* mice.

**
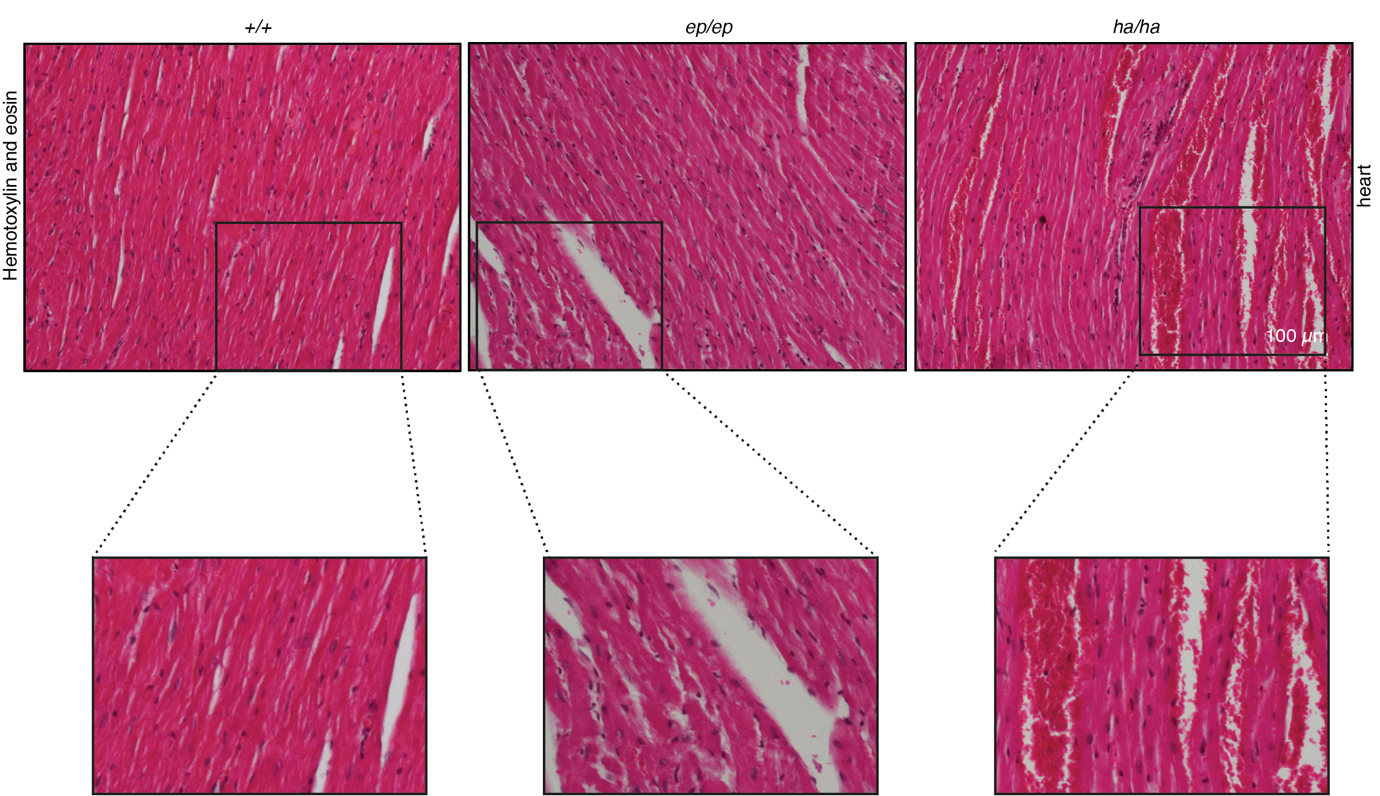
**

**Figure S2. Related to Figure 2.** Increased accumulation of blood in the heart of *Mrps12^ha/ha^* mice. Heart sections from *Mrps12^+/+^, Mrps12 ^ep/ep^* and *Mrps12^ha/ha^* mice fed a high-fat diet were cut to 5 µm thickness and stained with H&E.

**
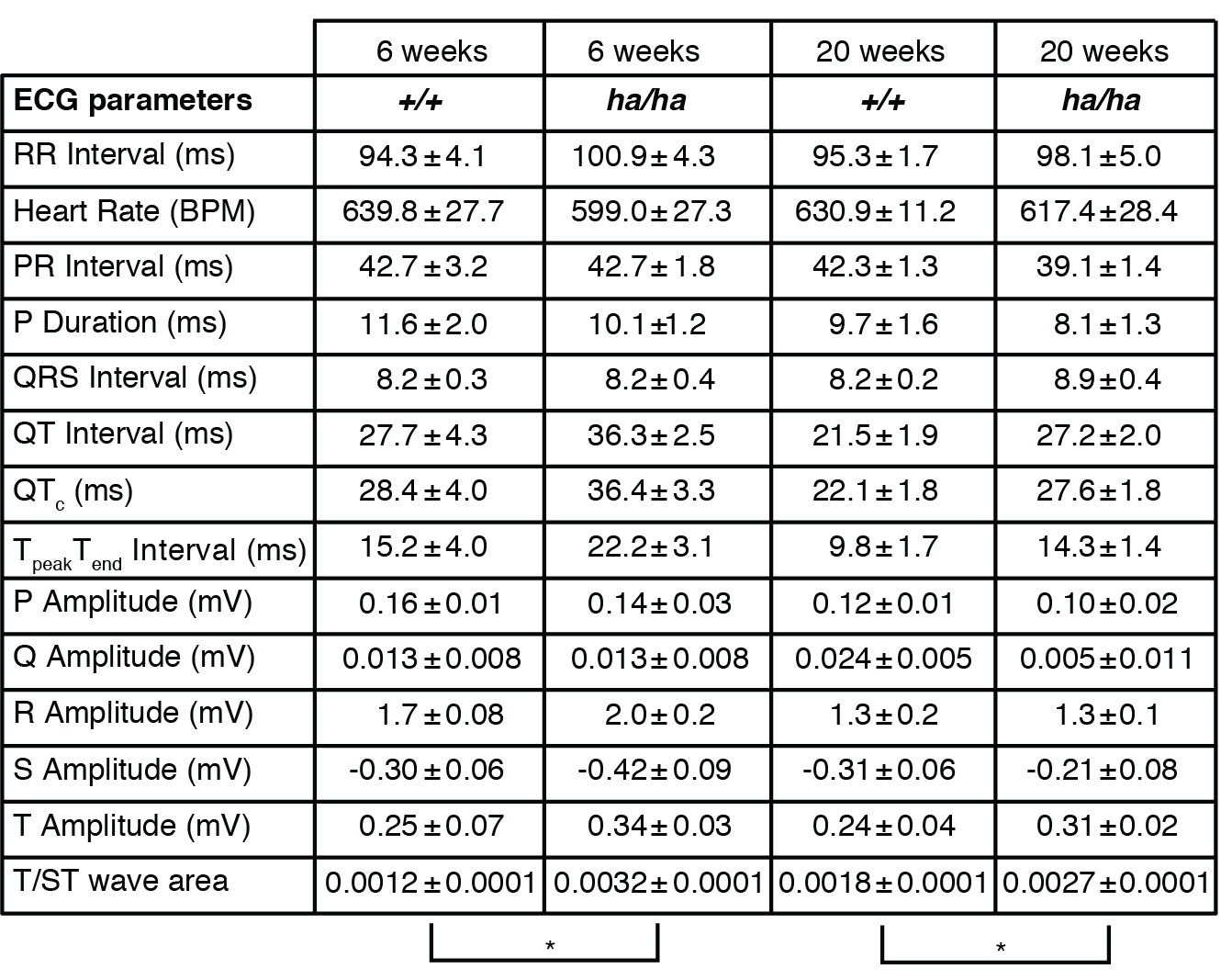
**

**Figure S3. Related to Figure 2.** Electrocardiographic measurements. Electrocardiographic recordings from *Mrps12^ha/ha^* (n=5) compared to *Mrps12^+/+^* mice (n=5) fed either a normal diet at six weeks of age or following a high-fat diet by 20 weeks of age. Values are means ± SEM. **p* < 0.05 compared with *Mrps12^+/+^*, Student’s t test.

**
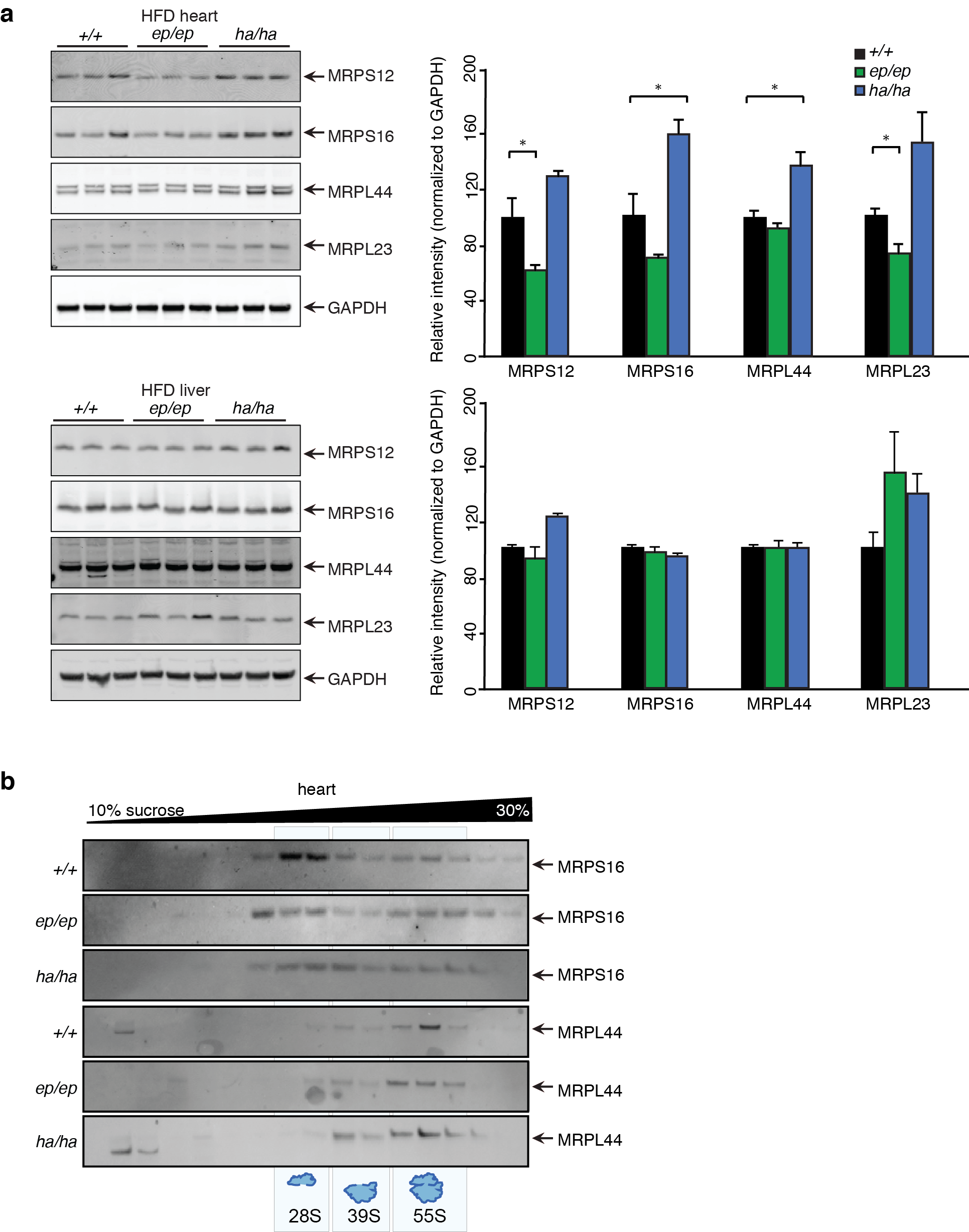
**

**Figure S4. Related to Figure 5.** The stability of the mitochondrial ribosome is not affected by metabolic stress. **(a)** Mitochondrial ribosomal protein abundance was measured by immunoblotting of heart or liver mitochondrial proteins isolated from 20-week-old *Mrps12^+/+^, Mrps12^ep/ep^* and *Mrps12^ha/ha^* mice fed a HFD. GAPDH was used as a loading control. Relative abundance of proteins was analysed using Li-Cor Odyssey Classic software relative to the loading control. Images are representative of blots from n=6 of each genotype. Values are means ± SEM. * *p* < 0.05, Student’s t test. **(b)** A continuous sucrose gradient was used to determine distribution of mitochondrial ribosomal protein markers of the small (MRPS16) and large (MRPL44) subunits in heart mitochondria isolated 20-week-old *Mrps12^+/+^, Mrps12^ep/ep^* and *Mrps12^ha/ha^* mice fed a HFD. All data are representative of results obtained from three independent biological experiments using at least six mice from each genotype.

**
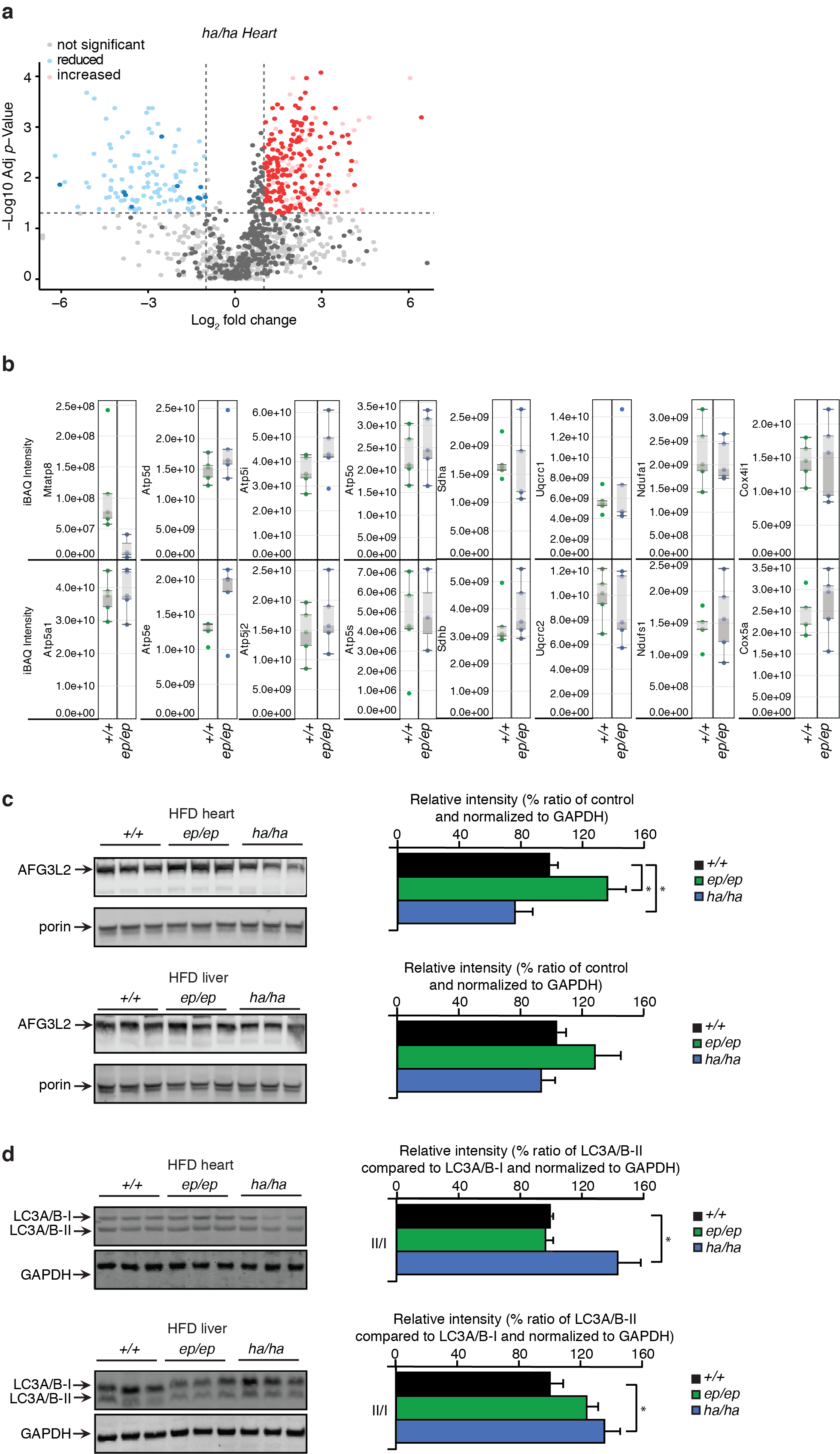
**

**Figure S5. Related to Figure 5.** The effects of altered fidelity of mitochondrial translation on mitochondrial protein stability, protease levels and autophagy. **(a)** Quantitative proteomic analysis of heart mitochondrial proteins from 20-week-old *Mrps12^ha/ha^* mice fed a HFD relative to their respective controls on a normal diet. The significant changes are shown in blue and red and the mitochondrial proteins are highlighted in dark blue, dark red and dark grey. **(b)** Proteomic quantification of electron transport chain proteins in isolated heart mitochondria from hearts of *Mrps12^+/+^* (WT, n=5), and *Mrps12^ep/ep^* (EP, n=5) animals, fed a HFD. Error bars represent 1.5 times the interquartile range. The mitochondrially encoded protein Atp8 was specifically reduced in the *Mrps12^ep/ep^* mice compared to *Mrps12^+/+^* mice fed a high-fat diet, showing that mistranslation destabilizes this protein. **(c)** Mitochondrial proteins from the heart and liver of *Mrps12^+/+^, Mrps12 ^ep/ep^* and *Mrps12^ha/ha^* mice fed a high-fat diet were immunoblotted to investigate the steady state levels of AFG3L2. Porin was used as a loading control and the relative abundance was analysed using Li-Cor Odyssey Classic software normalized to the loading control. Images are representative of blots from n=6 of each genotype. Values are means ± SEM. **p* < 0.05 Student’s *t* test. **(d)** The cellular levels of the short and long from of LC3A/B were measured in hearts and livers by immunoblotting from *Mrps12^+/+^, Mrps12 ^ep/ep^* and *Mrps12^ha/ha^* mice fed a a high-fat diet. GAPDH was used as a loading control and relative abundance of the proteins was analysed using Li-Cor Odyssey Classic software normalized to the loading control. Images are representative of blots from n=6 of each genotype. Values are means ± SEM. **p* < 0.05 Student’s *t* test.

**
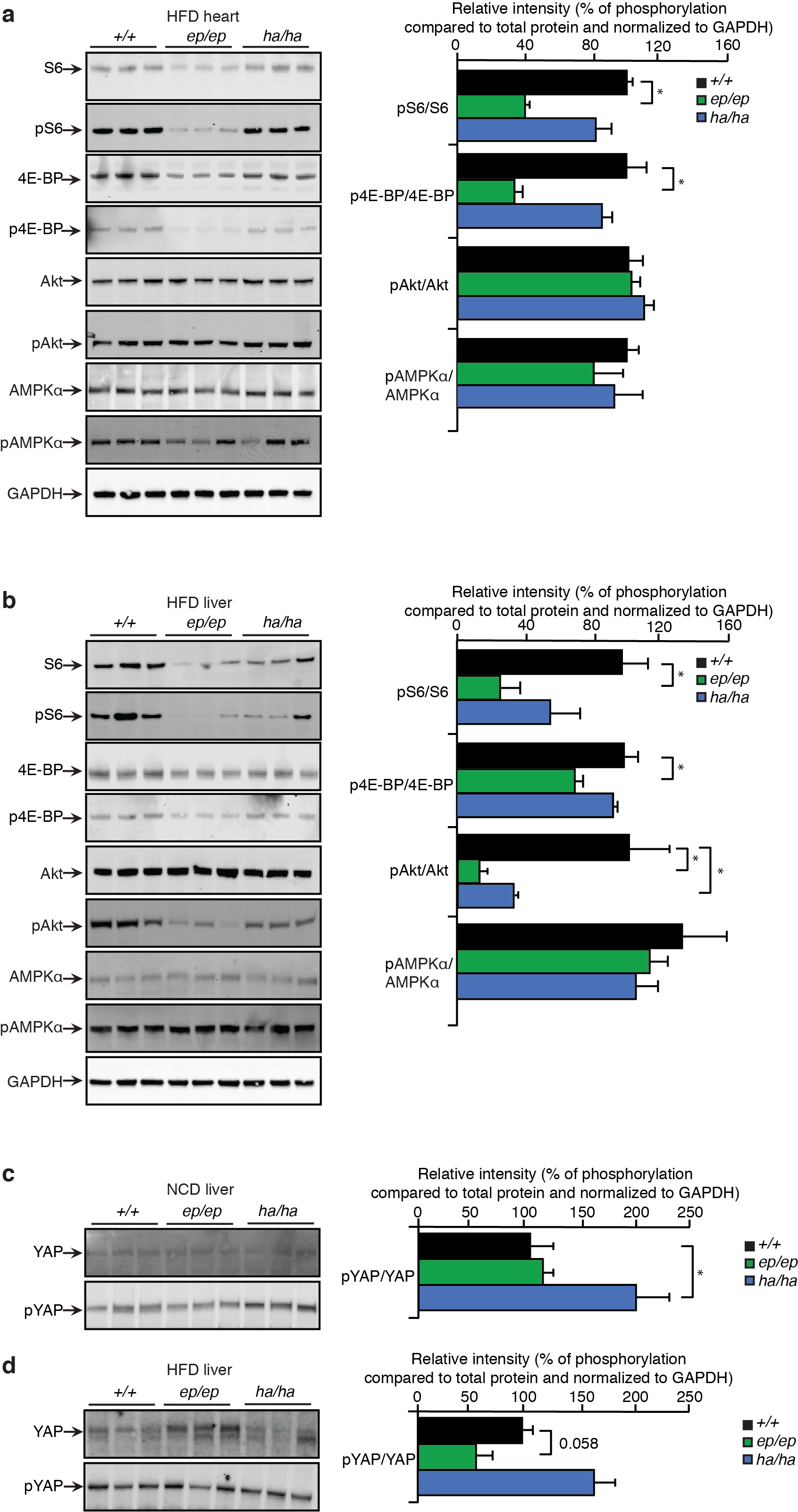
**

**Figure S6. Related to Figure 5.** The effects of altered translational fidelity on protein signalling. Cellular proteins from the heart **(a)** and liver **(b)** of *Mrps12^+/+^, Mrps12 ^ep/ep^* and *Mrps12^ha/ha^* mice fed a high-fat diet were immunoblotted to investigate the steady state and phosphorylated forms of S6, 4E-BP, Akt, and AMPKα. GAPDH was used as a loading control and the relative abundance of the phosphorylated proteins was analysed using Li-Cor Odyssey Classic software normalized to total protein, relative to the loading control. Images are representative of blots from n=6 of each genotype. Values are means ± SEM. * *p* < 0.05 Student’s t test. Abundance of steady state and phosphorylated forms of the transcriptional co-activator YAP1 were measured by immunoblotting against liver tissue from *Mrps12^+/+^, Mrps12 ^ep/ep^* and *Mrps12^ha/ha^* mice fed a normal **(c)** or a high-fat diet **(d)**. GAPDH was used as a loading control and relative abundance of the phosphorylated proteins was analysed using Li-Cor Odyssey Classic software normalized to total protein, relative to the loading control. Images are representative of blots from n=6 of each genotype. Values are means ± SEM. **p* < 0.05 Student’s t test.

**
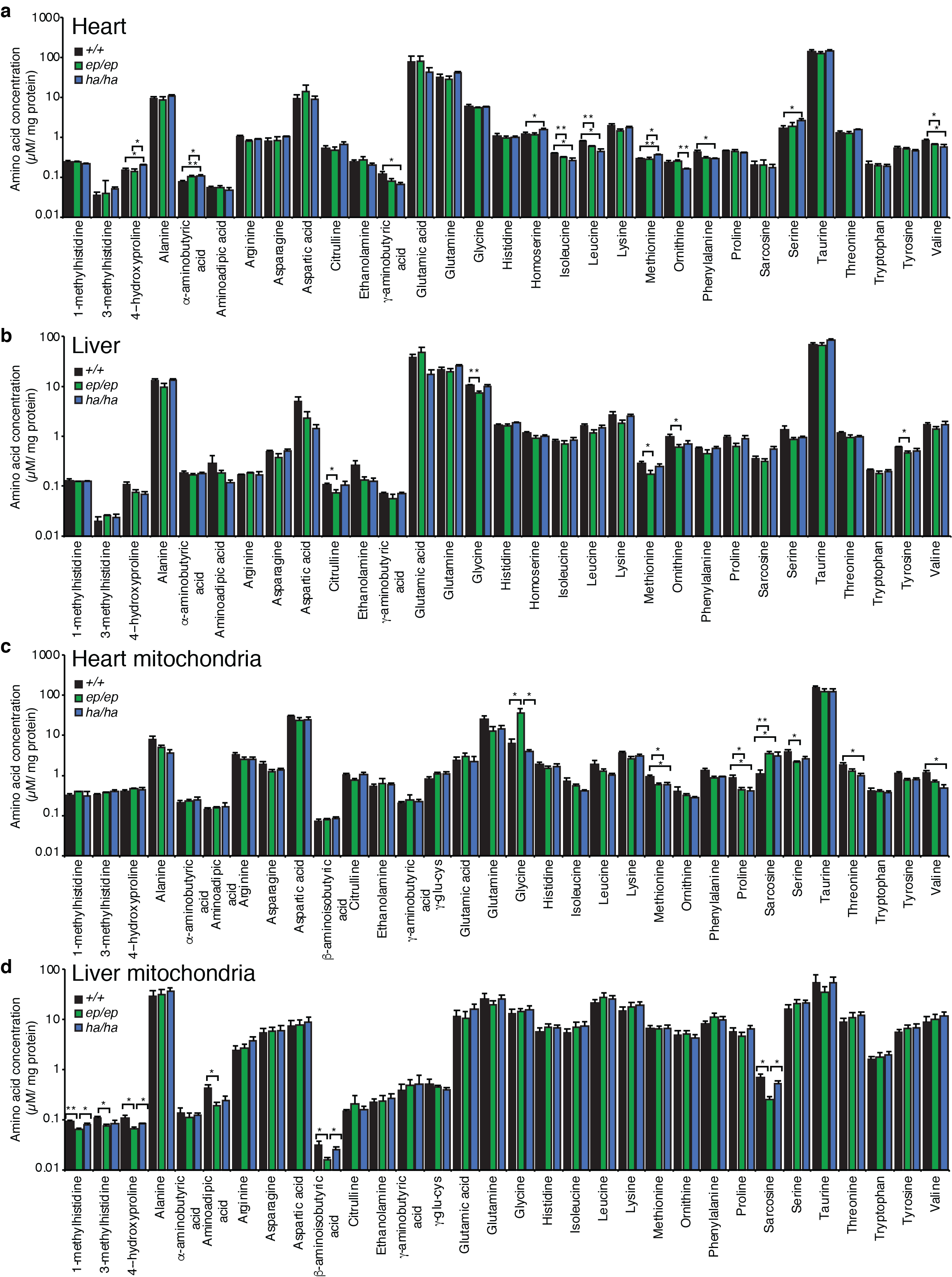
**

**Figure S7. Related to Figure 6.** Altering the fidelity of mitochondrial translation causes changes in the levels of specific amino acid under metabolic stress. Amino acid content was measured in hearts **(a)**, livers **(b)**, isolated heart mitochondria **(c)** and isolated liver mitochondria **(d)** from *Mrps12^+/+^* (n=5), *Mrps12^ep/ep^* (n=5) and *Mrps12^ha/ha^* mice (n=5), fed a HFD. Values are means ± SEM. **p* < 0.05, ***p* < 0.01 compared with *Mrps12^+/+^* using a Student’s *t* test.

**Supplemental Methods**

- 1. **Electrocardiography and echocardiography**

Echocardiograms were recorded using i13L probe on a Vivid 7 Dimension (GE Healthcare) as previously described (Ferreira et al. 2019). Measurements of left ventricular end-diastolic diameter (LVEDD), left ventricular end-systolic diameter (LVESD), fractional shortening (FS), left ventricular posterior wall in diastole (LVDPW), left ventricular posterior wall in systole (LVSPW), intraventricular septum in diastole (IVDS), intraventricular septum in systole (IVSS) and end-diastolic diameter (EDD) were made in M mode images recorded at a sweep speed of 200 mm.s^−1^, in triplicate from separate mice. Fractional shortening was calculated by the formula [(LVEDD − LVESD)/EDD] × 100.

- 1. **Histology**

Hearts were fixed with 10% neutral buffered formalin for 24 hours, washed in phosphate buffered saline and stored in 70% ethanol. Tissues were embedded in paraffin and sectioned using a microtome before transferring to positively charged slides. After heating the slides for 2 hours at 60˚C they were treated with xylene, xylene and ethanol (1:1) and ethanol at decreasing concentrations of 100%, 95%, 80%, and 60% before washing in distilled H_2_O. Freshly isolated livers were frozen in Optimal Cutting Temperature (OCT) medium and sectioned using a Leica, Cryostat 3050S. The H&E staining was performed as described before (Richman et al. 2015). Coverslips were attached using DPX mounting media (Scharlau) and images were acquired using a Nikon Ti Eclipse inverted microscope using a Nikon 20x objective.

- 1. **Transcriptomics**

RNA sequencing was performed on total RNA from three *Mrps12*^+/+^, three *Mrps12^ep/ep^* and three *Mrps12^ha/ha^* mice fed a high-fat diet. Sequencing was performed using the Illumina HiSeq platform, according to the Illumina Tru-Seq protocol and as we have done previously (Perks et al. 2018; Siira et al. 2018; Rackham et al. 2016). Sequenced reads were trimmed via TrimGalore v0.4.1 using cutadapt v1.18 (Krueger 2015; Martin 2011) and FastQC (Wingett & Andrews 2018) v0.11.8 in paired-end mode. Biologically independent replicates were pooled and gene expression was quantified with Salmon (Wingett & Andrews 2018) v1.1.0 using the selective alignment procedure with the GENCODE vM21 gene annotation and transcript sequences on the GRCm38.p6 (GRCm38.p6 genome assembly was downloaded from the EMBL-EBI FTP site ftp://ftp.ebi.ac.uk/pub/databases/gencode/Gencode_mouse/release _M17/GRCm38.p6.genome.fa.gz) genome sequence, adjusting for sequence and gc-content bias (-l ISR --seqBias --gcBias --validateMappings --mimicBT2). Differential expression analysis was performed on a per-tissue basis (excluding mitochondrial transcripts) with DESeq2 (Love et al. 2014) using counts summarised by tximport (Soneson et al. 2015) and effect size estimation performed using apeglm (Zhu et al. 2019) within the *lfcShrink* function. Functional analysis was performed with Gene Set Enrichment Analysis (Subramanian et al. 2005) v4.0.3 for each tissue and genotype under a HFD condition using default parameters with the KEGG gene set from MSigDB (Liberzon et al. 2011) and pre-ranked lists of log_2_ fold changes.

- 1. **Proteomics**

Proteomics was carried out on mitochondria isolated from hearts or livers from five *Mrps12*^+/+^, *Mrps12^ep/ep^* and *Mrps12^ha/ha^* mice fed either a chow or high-fat diet at 20 weeks. Mitochondrial proteins (100 µg) were resuspended in lysis buffer (6 M guanidinium chloride, 2.5 mM tris (2-carboxyethyl) phosphine hydrochloride, 10 mM chloroacetamide, and 100 mM Tris-HCl). After lysis, samples were diluted 1:10 in 20 mM Tris-HCL pH 8.0 and 100 µg of protein were mixed with 1 µg of Trypsin Gold (Promega) and incubated overnight at 37˚C to achieve complete digestion. Peptides were cleaned with home-made STAGEtips (Empore Octadecyl C18; 3M, Germany) and eluted in 60% acetonitrile/0.1% formic acid buffer. Samples were dried in a SpeedVac apparatus (Eppendorf concentrator plus 5305) at 45˚C and the peptides were suspended with 0.1% formic acid, 1.5 µg of peptides were analyzed by LC- MS/MS. For mass spectrometric analysis, peptides were separated on a 50 cm long, 75 µm internal diameter EASY-spray PepMap C18 column (Thermo Fisher Scientific) using a Dionex (Ferreira et al. 2019). The Proteome Discoverer (PD, v.2.3.0.523; Thermo Scientific) software was used to process raw (Xcalibur) label-free MS/MS data produced by the Orbitrap Fusion (v.3.0.2041) mass spectrometer as described previously (Ferreira et al. 2019). Protein abundance results were visualised with OmicsVolcano (Kuznetsova et al. 2021).

Follow-up proteomic analysis was performed on the mitochondria isolated from heart tissues. Samples were processed as described in (Harney et al. 2021), with the exception that chloroform-methanol precipitation was not used. Briefly, mitochondria were lysed in 4% sodium deoxycholate (SDC), 100mM tris-HCl (pH 8.5) with immediate heating of the lysates to 95°C for at least 10 min. Protein concentration was determined by BCA total protein assay (Pierce). 20ug of protein was reduced with 10mM TCEP and alkylated with 40mM chloroacetamide simultaneously at 95°C for 10 min. Lysates were then diluted to a final concentration of 1% SDC using MQ water and digested overnight with 400ng MS-grade trypsin (in 50mM acetic acid) at 37°C with constant rocking. Samples were diluted with ethyl acetate (50% final concentration, v/v) and vortexed until all the precipitated SDC was resuspended. StageTips were prepared and sample purification performed with SDB-RPS StageTips. Peptides were reconstituted with 5% formic acid in MS-grade water, sealed and stored at 4°C until LC-MS/MS acquisition. Peptide samples prepared as in Trypsin digestion for proteome analysis, were directly injected onto a 50cm x 70 um C18 (Dr. Maisch, Ammerbuch, Germany, 1.9 um) fused silica analytical column with a 10 μm pulled tip, coupled online to a nanospray ESI source. Peptides were resolved over a gradient from 5% - 40% acetonitrile over 120 min with a flow rate of 300 nL min-1. Peptides were ionized by electrospray ionization at 2.3 kV. MS/MS analysis was performed using a Thermo Eclipse Tribrid mass spectrometer (Thermo Fisher) with HCD fragmentation. Spectra were attained in a data-dependent acquisition of the top 20 most abundant ions at any individual point during the gradient. RAW data were analyzed using the quantitative proteomics software MaxQuant (https://www.maxquant.org). Peptide and protein level identification were both set to a false discovery rate of 1% using a target-decoy based strategy. The database supplied to the search engine for peptide identifications contained both the mouse UniProt database and a custom-made mistranslation database for mitochondrially encoded proteins. Mass tolerance was set to 4.5 ppm for precursor ions and MS/MS mass tolerance was 20 ppm. Enzyme specificity was set to trypsin (cleavage C-terminal to Lys and Arg) with a maximum of 2 missed cleavages permitted. Deamidation of Asn and Gln, oxidation of Met, pyro-Glu (with peptide N-term Gln) and protein N-terminal acetylation were set as variable modifications. Carbamidomethyl on Cys was searched as a fixed modification.

- 1. **Generation and testing of *C. elegans* mutants**

To generate the CRISPR-Cas9 mutant strains, young adult N2 worms were injected with the following mix: 50 ng/µL pDD162-*mrps-12* (*mrps-12 -* sgRNA), 50 ng/µL pDD162-*dpy-10* (*dpy-10* – sgRNA), 10 ng/µL pGH8 (*rab-3p::mCherry*), 5 ng/µL pCFJ104 (*myo-3p::mCherry*), 2.5 ng/µL pCFJ90 (*myo-2p::mCherry*), 10 pmol *dpy-10* ssODN (repair template). For SJZ819 [*MRPS-12(K89T*)] 10 pmol of K89T ssODN was added to the above mix and for SJZ820 [*MRPS-12(K90I*)] 10 pmol of K90I ssODN was added. pDD162-*mrps-12*(*mrps-12*::sgRNA) was generated using reverse PCR-based amplification to yield a sg-RNA with the sequence CACCCCAAAAAGCCAAACUC.

pDD162-*dpy-10*(*dpy-10*::sgRNA) was generated using reverse PCR-based amplification on the template vector pDD162 to yield a sgRNA with the sequence GCUACCAUAGGCACCACGAG. The ssODN repair template for both mutants also introduced a silent EcoRI site to facilitate subsequent genotyping. Subsequent selection steps were performed as described previously (Paix et al. 2017).

Total RNA (500 ng-1000 ng) was reverse transcribed using the ProtoScript II reverse transcription kit (NEB) for 1 h at 42°C followed by enzyme inactivation at 95°C for 5 min. Quantitative reverse transcriptase PCR (qRT-PCR) was performed using a LightCycler 480 II (Roche) with SensiFAST SYBER No-ROX (Bioline meridian Biosystems) using gene specific primers hsp-6-F CAAACTCCTGTGTCAGTATCATGGAAGG, hsp-6-R GCTGGCTTTGACAATCTTGTATGGAACG, tba-1-F TCAACACTGCCATCGCCGCC, and tba-1-R TCCAAGCGAGACCAGGCTTCAG. PCR was carried out in technical triplicates using the following cycling conditions; 95°C for 3 min, followed by 45 cycles of denaturation at 95°C for 10 s, annealing at 60°C for 10 s and elongation at 72°C for 20 s. A melting curve was generated at the end of the amplification in every run to confirm primer specificity. Threshold cycle (C_t_) values were determined by calculating the second derivative maximum of three technical triplicates for each sample.

Lifespan assays were carried out essentially as described previously (Amrit et al. 2014). Briefly ~100-120 L4 stage worms from the indicated genotypes were picked onto 10 individual plates seeded with OP50 (10-12 worms per plate representing Day 0). Plates were subsequently grown at 20^o^C for the duration of the experiment. Worms were manually transferred every two days until such stage as egg laying had ceased. Surviving worms were counted and recorded every two days, with worms ‘censored’ if they didn’t meet specific criteria as described previously (Amrit et al. 2014).

**References:**

Amrit FRG, Ratnappan R, Keith SA & Ghazi A (2014) The C. elegans lifespan assay toolkit. *Methods* 68, 465–475.

Ferreira N, Perks KL, Rossetti G, Rudler DL, Hughes LA, Ermer JA, Scott LH, Kuznetsova I, Richman TR, Narayana VK, Abudulai LN, Shearwood A-MJ, Cserne Szappanos H, Tull D, Yeoh GC, Hool LC, Filipovska A & Rackham O (2019) Stress signaling and cellular proliferation reverse the effects of mitochondrial mistranslation. *The EMBO Journal* 313, e102155.

Harney DJ, Cielesh M, Chu R, Cooke KC, James DE, Stöckli J & Larance M (2021) Proteomics analysis of adipose depots after intermittent fasting reveals visceral fat preservation mechanisms. *CellReports* 34, 108804.

Krueger F (2015) Trim Galore: A wrapper tool around Cutadapt and FastQC. *httpwww.bioinformatics.babraham.ac.ukprojectstrimgalore*.

Kuznetsova I, Lugmayr A, Rackham O & Filipovska A (2021) OmicsVolcano: software for intuitive visualization and interactive exploration of high-throughput biological data. *STAR Protocols* 2, 100279.

Liberzon A, Subramanian A, Pinchback R, Thorvaldsdóttir H, Tamayo P & Mesirov JP (2011) Molecular signatures database (MSigDB) 3.0. *Bioinformatics* 27, 1739–1740.

Love MI, Huber W & Anders S (2014) Moderated estimation of fold change and dispersion for RNA-seq data with DESeq2. *Genome Biology* 15, 550.

Martin M (2011) Cutadapt removes adapter sequences from high-throughput sequencing reads. *EMBnet journal* 17, 10–12.

Paix A, Folkmann A, Goldman DH, Kulaga H, Grzelak MJ, Rasoloson D, Paidemarry S, Green R, Reed RR & Seydoux G (2017) Precision genome editing using synthesis-dependent repair of Cas9-induced DNA breaks. *Proc Natl Acad Sci USA* 114, E10745–E10754.

Perks KL, Rossetti G, Kuznetsova I, Hughes LA, Ermer JA, Ferreira N, Busch JD, Rudler DL, Spåhr H, Schöndorf T, Shearwood A-MJ, Viola HM, Siira SJ, Hool LC, Milenkovic D, Larsson N-G, Rackham O & Filipovska A (2018) PTCD1 Is Required for 16S rRNA Maturation Complex Stability and Mitochondrial Ribosome Assembly. *CellReports* 23, 127–142.

Rackham O, Busch JD, Matic S, Siira SJ, Kuznetsova I, Atanassov I, Ermer JA, Shearwood A-MJ, Richman TR, Stewart JB, Mourier A, Milenkovic D, Larsson N-G & Filipovska A (2016) Hierarchical RNA Processing Is Required for Mitochondrial Ribosome Assembly. *Cell Reports* 16, 1874–1890.

Richman TR, Rackham O, Ermer JA, Davies SMK, Perks KL, Viola HM, Filipovska A, Shearwood A-MJ & Hool LC (2015) Mutation in MRPS34 Compromises Protein Synthesis and Causes Mitochondrial Dysfunction. *PLoS Genet* 11, e1005089.

Siira SJ, Rossetti G, Richman TR, Perks K, Ermer JA, Kuznetsova I, Hughes L, Shearwood A-MJ, Viola HM, Hool LC, Rackham O & Filipovska A (2018) Concerted regulation of mitochondrial and nuclear non-coding RNAs by a dual-targeted RNase Z. *EMBO Rep* 19, e46198–18.

Soneson C, Love MI & Robinson MD (2015) Differential analyses for RNA-seq: transcript-level estimates improve gene-level inferences. *F1000Res* 4, 1521.

Subramanian A, Tamayo P, Mootha VK, Mukherjee S, Ebert BL, Gillette MA, Paulovich A, Pomeroy SL, Golub TR, Lander ES & Mesirov JP (2005) Gene set enrichment analysis: a knowledge-based approach for interpreting genome-wide expression profiles. *Proc Natl Acad Sci U S A* 102, 15545–15550.

Wingett SW & Andrews S (2018) FastQ Screen: A tool for multi-genome mapping and quality control. *F1000Res* 7, 1338.

Zhu A, Ibrahim JG & Love MI (2019) Heavy-tailed prior distributions for sequence count data: removing the noise and preserving large differences. O. Stegle, ed. *Bioinformatics* 35, 2084–2092.
